# Par1b Induces Asymmetric Inheritance of Plasma Membrane Domains via LGN-Dependent Mitotic Spindle Orientation in Proliferating Hepatocytes

**DOI:** 10.1371/journal.pbio.1001739

**Published:** 2013-12-17

**Authors:** Christiaan L. Slim, Francisco Lázaro-Diéguez, Marjolein Bijlard, Mathilda J. M. Toussaint, Alain de Bruin, Quansheng Du, Anne Müsch, Sven C. D. van IJzendoorn

**Affiliations:** 1 Department of Cell Biology, University Medical Center Groningen, University of Groningen, Groningen, The Netherlands; 2 Department of Developmental and Molecular Biology, Albert Einstein College of Medicine, Bronx, New York, United States of America; 3 Department of Pathobiology, Faculty of Veterinary Medicine, Utrecht University, Utrecht, The Netherlands; 4 Institute of Molecular Medicine and Genetics, Department of Neurology, Medical College of Georgia, Georgia Regents University, Augusta, Georgia, United States of America; Dana-Farber Cancer Institute, United States of America

## Abstract

The development and maintenance of polarized epithelial tissue requires a tightly controlled orientation of mitotic cell division relative to the apical polarity axis. Hepatocytes display a unique polarized architecture. We demonstrate that mitotic hepatocytes asymmetrically segregate their apical plasma membrane domain to the nascent daughter cells. The non-polarized nascent daughter cell can form a de novo apical domain with its new neighbor. This asymmetric segregation of apical domains is facilitated by a geometrically distinct “apicolateral” subdomain of the lateral surface present in hepatocytes. The polarity protein partitioning-defective 1/microtubule-affinity regulating kinase 2 (Par1b/MARK2) translates this positional landmark to cortical polarity by promoting the apicolateral accumulation of Leu-Gly-Asn repeat-enriched protein (LGN) and the capture of nuclear mitotic apparatus protein (NuMA)–positive astral microtubules to orientate the mitotic spindle. Proliferating hepatocytes thus display an asymmetric inheritance of their apical domains via a mechanism that involves Par1b and LGN, which we postulate serves the unique tissue architecture of the developing liver parenchyma.

## Abstract 2

Proliferating hepatocytes in the liver show an atypical, asymmetric mode of cell division, which is coordinated by Par1b and LGN and may explain the unique tissue architecture of the liver.

## Author Summary

The development and maintenance of the polarized epithelial architecture and function of organs that form tubular “lumen” structures is important for normal physiology and, when deregulated, gives rise to disease. Recent studies have highlighted the importance of a strict coordination of the orientation of mitotic divisions relative to an internal axis of asymmetry in proliferating epithelial cells during this process. Hepatocytes are the predominant epithelial cells of the liver. Hepatocytes display a unique lumen-forming architecture and cellular asymmetry, but the molecular basis for this special polarized architecture is not well understood. Our study now reveals an unexpected mode of plasma membrane domain inheritance that is coupled to a cellular axis of asymmetry in proliferating mammalian hepatocytes. We show that mitotic hepatocytes asymmetrically segregate their apical plasma membrane (the membrane facing the lumen structure) along with the lumen to their daughter cells. We demonstrate that the coordinated action of two proteins, Par1b and LGN, constitutes a fundamental part of the underlying molecular mechanism. This coupling of cell division and polarity in hepatocytes is distinct from that established in other epithelial cell types. These findings are important for understanding the unique polarized tissue architecture in the developing liver.

## Introduction

The liver is a vital organ. Hepatocytes occupy more than 85% of the parenchymal liver mass and are responsible for a wide range of biological processes. These include the synthesis of plasma proteins and the processing of nutrients and toxic compounds from the blood that passes through the liver sinusoids. Hepatocytes also produce and secrete bile. Bile contributes to fat emulsion in the intestine and the elimination of detoxified compounds via the feces. Hepatocytes form a branching network of bile canaliculi between the cells that efficiently drains the secreted bile out of the liver parenchyme while keeping it separate from the blood [1],[2]. The microanatomy of this canalicular network is unique to the liver [3]. Defects in the bile canalicular network and bile flow are associated with liver diseases [4].

**Figure 1:**
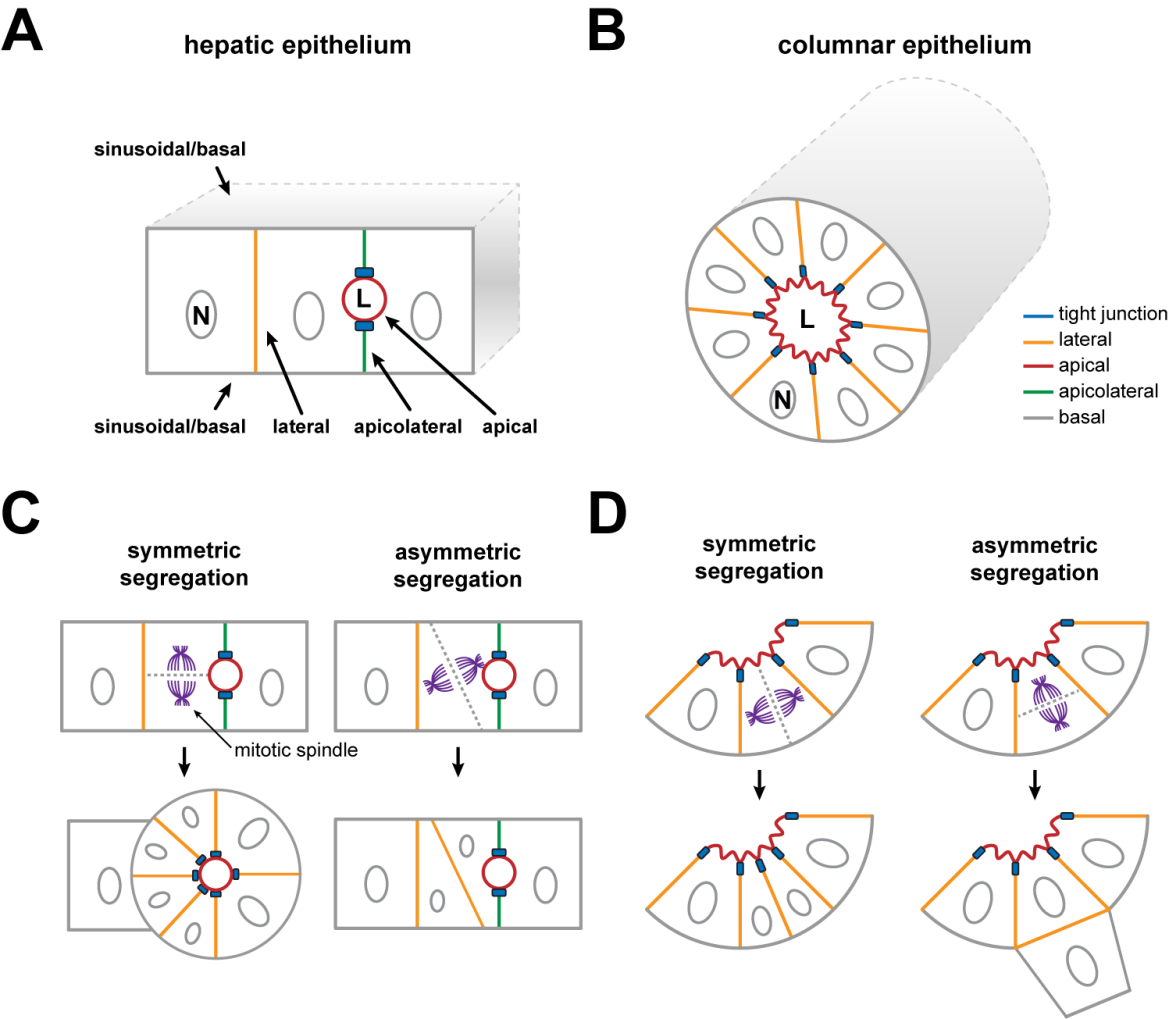
Schematic overview of the difference between columnar and hepatic epithelium. (A) Schematic overview of hepatic epithelium and polarity. Note the presence of an apicolateral plasma membrane domain in this type of epithelium. (B) Schematic overview of columnar (i.e., “simple”) epithelium and polarity. (C) Schematic representation of the tissue architecture resulting from symmetric and asymmetric segregation of the apical surface in hepatic epithelium. (D) Schematic representation of the tissue architecture resulting from symmetric and asymmetric segregation of the apical surface in columnar epithelium. The dashed line represents the cleavage furrow and the position of the newly formed membrane. L, lumen; N, nucleus.

Knowledge of the cell biological principles and molecular mechanisms that underlie the development of the bile canalicular network is limited. This is in part due to the lack of in vitro cell culture model systems that combine cell proliferation and canalicular network formation. Nevertheless, different in vitro cell model systems can reproduce specific steps in the process of bile canalicular network formation. For instance, from early microscopy studies of embryonic rat livers we know that the formation of isolated small spherical lumens between mitotically active hepatocytes is the first step in bile canalicular network development [5]–[8] (Figure 1A), and this process is reproduced by hepatic HepG2 [9],[10] and WIF-B9 [11] cell lines. Both in vivo and in vitro, the formation of these primordial intercellular lumens is accompanied by the segregation of the hepatocyte surface into a lumen-facing apical domain and a sinusoid-facing basal domain, each with a specific protein and lipid composition (Figure 1A) [7]–[11]. The establishment of cell surface domains is the hallmark of apical–basal cell polarity [12].

The early establishment of apical–basal polarity is instrumental for the functional shaping of a proliferating epithelial cell mass [13],[14]. Indeed, dividing cells not only generate enough critical cell mass to create the tissue, but they also make use of their apical–basal polarity axis (PA) to orientate their mitotic spindle apparatus [15]. By orientating its mitotic spindle apparatus, the dividing polarized epithelial cell can control the position of the emerging new nuclei, and hence the position of the daughter cells, relative to the position of the primordial apical domain and lumen. The same principles are used when dividing cells repair tissue damage [16].

The unique microanatomy of the bile canalicular network suggests that the mode of cell division orientation in hepatocytes—from the moment that they have established apical–basal polarity—differs from that observed in “simple” epithelial cells such as intestinal or kidney epithelial cells. Indeed, simple epithelial cells do not develop a canalicular network between cells. Instead, they develop large cystic lumens and tubes (Figure 1B) via a process that is dependent on a Leu-Gly-Asn repeat-enriched protein (LGN)–mediated orientation of the mitotic spindle apparatus that is strictly perpendicular to the apical–basal axis, and the resultant symmetric segregation of the apical domain to both daughter cells [17]–[19] (Figure 1D). A mitotic spindle orientation that is strictly perpendicular to the apical PA would in mitotic hepatocytes thus be predicted to promote the development of cystic lumens rather than of canalicular networks (Figure 1C).

In order to investigate the orientation of the mitotic spindle in hepatocytes within their native environment, immunohistochemistry can be performed on fixed liver tissues. To study the molecular regulation and the dynamics of mitotic spindle orientation and cell division in hepatocytes, cell lines are the best model of choice. In this study we have combined the analysis of liver tissues with that of HepG2 and WIF-B9 cell lines to investigate the relationship between cell polarity and the orientations of the mitotic spindle and cell division at the molecular level in hepatocytes.

## Materials and Methods

### Antibodies and Reagents

Commercial antibodies used for immunofluorescence are listed in Table S1. The rabbit anti-LGN antibody was described earlier [20]. Phalloidin-TRITC was used to label F-actin (P1951; Sigma). DAPI was from Invitrogen, and DRAQ5 was purchased from Cell Signaling Technology.

### Plasmids

The plasmid expressing H2B-mCherry was a kind gift from B. Giepmans (University Medical Center Groningen, the Netherlands). The plasmid expressing human Par1b was a kind gift from H. Miki (Osaka University, Japan). Overexpression was obtained by cloning constructs into a lentiviral expression system [21]. Briefly, constructs were cloned into pENTR1A (Addgene plasmid 17398) and recombined into pLenti-CMV-Puro (Addgene plasmid 17452) using LR clonase (Life Technologies). The Par1b-KD construct was described before [22].

### Cell Lines and Tissues

HepG2 cells were cultured as previously described [23]. HepG2 cells expressing ABCB1-eGFP were cultured as previously described [24]. For experiments, cells were plated on ethanol-sterilized glass coverslips at a density of 5×10^4^ cells/cm^2^ and grown for 2 d. For Par1b knockdown experiments, cells were plated at 15×10^4^ cells/cm^2^ to match WIF-B9 conditions (see below). WIF-B9 cells were grown in modified F-12 Coon's modification medium (F6636; Sigma) supplemented with 10^−6^ M hypoxanthine, 4×10^−8^ M aminopterin, 1.6×10^−6^ M thymidine, 5% (v/v) fetal bovine serum (100–106; Gemini), 1% glutamax (Invitrogen), 0.5 µg/ml amphotericin, and 10 mM HEPES. For culture maintenance, cells were seeded in plastic dishes at 10×10^3^ cells/cm^2^ and cultivated up to 4 d before replating. For experiments, differentiated cultures (10–12 d) were plated on water-prewashed glass coverslips (EMS) in MatTek chambers at 15×10^4^ cells/cm^2^. Madin-Darby canine kidney (MDCK) cells were grown in DMEM without phenol red (17–205; Cellgro) supplemented with 10% fetal bovine serum (S11050; Atlanta Biologicals) and 2 mM L-glutamine. Stable MDCK cell lines expressing gp135-GFP and Par1b were generated from T23-MDCK cells. MDCK-Par1b cells were prepared as previously described [22]. Cells were maintained at 37°C in a 5% CO_2_ (HepG2 and MDCK cells) or 7% CO_2_ (WIF-B9 cells) humidified atmosphere.

Mouse liver tissues 48 h after partial hepatectomy (formalin-fixed paraffin-embedded) and mouse liver tissue from 23-d-old mice, collected 2 d after weaning (prepared as near-native tissue slices as previously described [25]), were prepared as previously described [26]. Formalin-fixed paraffin-embedded rat liver tissue (collected 2 d after weaning) was a kind gift from C. Desdouets (INSERM, France).

### RNA Interference

For HepG2 cells, RNA interference was performed using the pLKO lentiviral knockdown system (http://www.addgene.org/tools/protocols/plko/). The target sequences used for Par1b and LGN are listed in Table S2 and were generated according to the pLKO protocol. Knockdown was verified by real-time PCR on a StepOnePlus system (Applied Biosystems) using the primers listed in Table S3. A short hairpin RNA (shRNA)–resistant Par1b was created by introducing missense mutations into the shRNA target sequence (AGCAAGAGAGGCACTTTA to AGTAAAAGGGGAACATTG) using a Q5 Site-Directed Mutagenesis Kit (E0554S; New England Biolabs). All constructs were verified by sequencing. RNA was isolated using Tri-Reagent from Sigma (T9424). RNA interference experiments in WIF-B9 and MDCK cells were performed as previously described [22].

### Virus Production and Transduction

Lentiviral particles were created using a second-generation system based on pCMVdR8.1 (structural components) and pVSV-G (envelope protein). Briefly, 2.6×10^6^ HEK293T cells were plated in a 10-cm dish. The next day, the cells were co-transfected with CaPO_4_-DNA complexes of pCMVdR8.1, pVSV-G, and either pLKO.1 or pLenti constructs for ∼16 h. Medium was refreshed, and after 24 and 48 h viral particles were harvested and filtered through a 0.45-µm PVDF membrane filter. Viral supernatants were stored at −80°C. 1-d-old HepG2 cells were infected with viral particles for 24 h, whereafter cells were incubated with normal growth medium to recover from the viral infection. Selection medium (1 µg/ml puromycin; Sigma) was added 24 h later to select for transduced cells. WIF-B9 cells expressing DPPIV-TagRFP, GFP, Par1b-DN-GFP, pSUPER-GFP, or shRNA Par1b-GFP were obtained by adenovirus-mediated transduction [22] in Opti-MEM (Invitrogen) for 1 h with one plaque-forming unit/cell and 10–12 h expression at 37°C.

### Immunofluorescence and Microscopy

Cells were fixed in 4% paraformaldehyde at 37°C (MDCK and WIF-B9 cells) or for 20 min at room temperature (HepG2 cells). For staining microtubular structures in HepG2 cells (tubulin, NuMA, and LGN), cells were pre-extracted in 0.5% Triton X-100 in PHEM buffer (1 min), washed once in PHEM buffer, and fixed in 4% paraformaldehyde in PHEM buffer. For HepG2 cells, blocking and permeabilization were performed for 30 min at room temperature in HBSS containing 0.025% saponin (w/v), 1% (w/v) BSA, and 0.02% sodium azide, followed by antibody staining in the same buffer. WIF-B9 and MDCK cells were permeabilized with 0.2% Triton X-100 and blocked with 1% BSA. Antibody incubation was performed in PBS–1% BSA.

HepG2 cells were imaged using a combination of widefield (Olympus AX70) and confocal microscopy (Leica SP2; HCX PL APO 63x/1,4 oil; pinhole 1 AU; pixel size 80 nm) and analyzed using a combination of Imaris (Bitplane), ImageJ, and Adobe Photoshop CS4. A Solamere Nipkow confocal live cell imaging system was used (HCX PL APO 63x/1,3 glycerin; pixel size 117 nm) to live-image *z*-stacks of 7×1.5 µm every 4 min, unless otherwise indicated. WIF-B9/MDCK cells were imaged with a TCS SP5 confocal microscope (Leica Microsystems) equipped with a motorized *x-y* stage for multiple position finding and with an 8,000-Hz resonant scanner. Fixed cells were imaged using a HCX PL APO 63x/1.4-0.60 oil λBL CS objective on glass coverslips mounted in non-hardening, glycerol-based aqueous mounting medium. Confocal (pinhole 1 AU; pixel size 80.02 nm) *xyz*-stacks were taken from the monolayer. Live cell imaging was performed using a HCX PL APO 40x/1.25-0.75 oil CS objective on MatTek or CELLview chambers. *xyzt*-stack frames (pinhole 2–3 AU; pixel size 100.1–252.8 nm) were recorded. Image analysis was performed using LAS AF 2.3.1 and ImageJ 1.45 software. Brightness and contrast were adjusted according to the *Journal of Cell Biology* guidelines, without changing gamma settings.

### Calculations and Statistics

For calculating the orientation of the mitotic spindle in cell lines, a line was drawn from the center of the apical lumen through the center of the mitotic spindle (PA). A second line was drawn through the spindle poles (spindle axis [SA]). When no spindle pole staining was performed, it was assumed that the spindle poles were localized in a straight line perpendicular to the metaphase plate. The angle between these lines (SA/PA) was calculated with the ImageJ measure angle tool and plotted accordingly. To study the orientation of cell division in rat and mouse liver tissue, a line was drawn through both spindle poles of a dividing cell and extrapolated to determine the plasma membrane domain to which the spindle poles were oriented. The orientation of the spindle poles was scored as oriented towards (1) the bile canaliculus, (2) the apicolateral domain, or (3) the basolateral (sinusoidal) or common lateral membrane. Microsoft Excel was used for calculations, and Graphpad PRISM was used to generate graphs. Graphs represent mean ± standard deviation of three independent experiments, unless otherwise specified. Sample sizes (*n*) in graphs represent the total sample size. The statistical significance of differences was determined using Student's *t*-test (two-tailed, unpaired, with equal variance) unless otherwise specified.

## Results

### The Mitotic Spindle in Hepatocytes In Vivo Is Orientated towards an LGN-Enriched Apicolateral Plasma Membrane Domain

**Figure 2:**
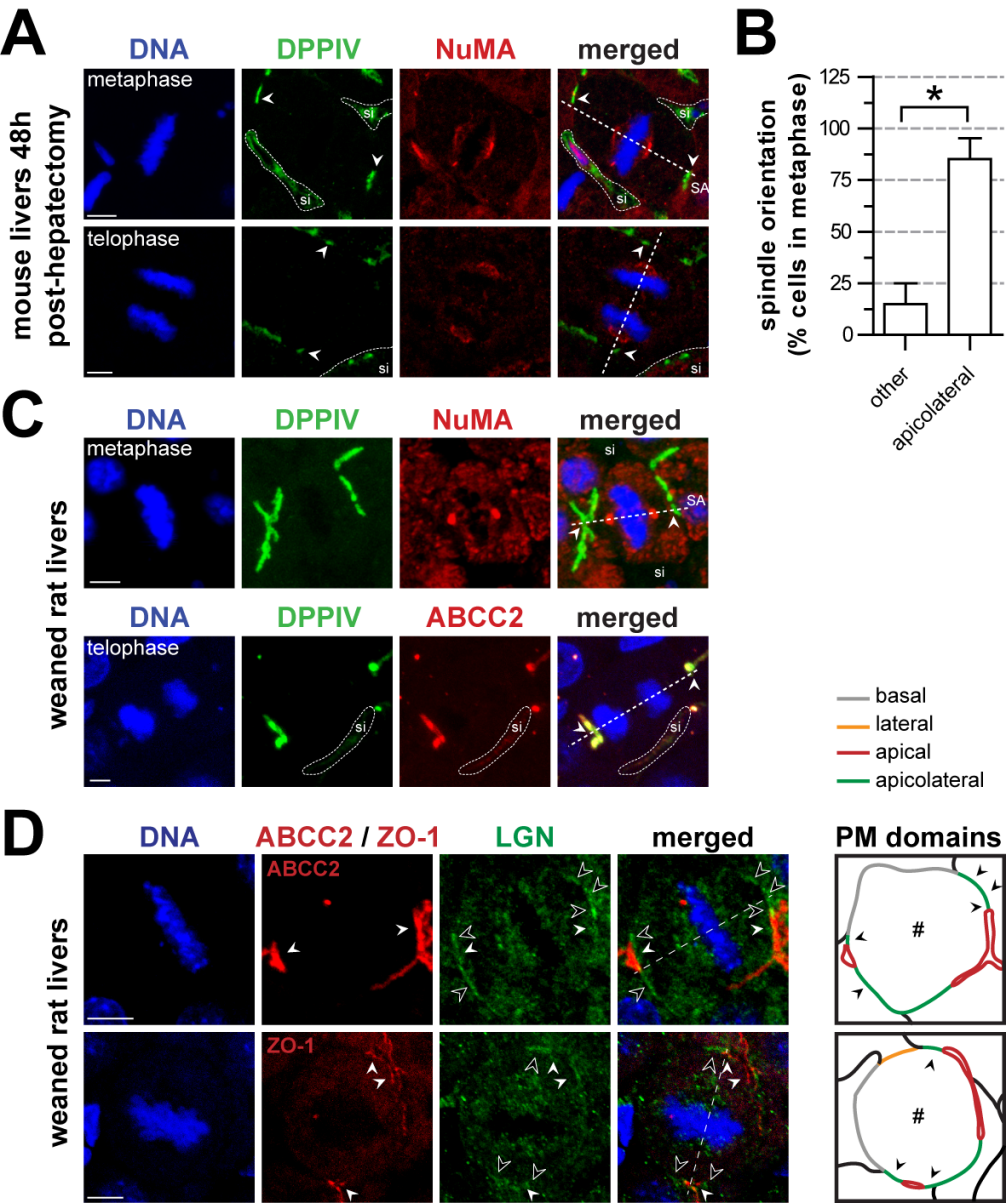
Rat and mouse hepatocytes predominantly orient their mitotic spindle axis towards the apicolateral subdomain. (A) Hepatocytes from mouse livers 48 h post-hepatectomy orient their spindle poles (labeled with NuMA) towards the apicolateral subdomain. (B) Quantification of (A) (*n* = 61). Dividing hepatocytes predominantly orient their SA towards the apicolateral subdomain. (C) Dividing hepatocytes from weaned rat livers orient their spindle poles (marked by NuMA) towards the apicolateral subdomain (marked by DPPIV and ABCC2) in metaphase and telophase. (D) Apicolateral localization of LGN (white outline arrowheads) in dividing rat liver hepatocytes. The apical domain is labeled with ABBC2. Tight junctions are labeled with ZO-1. The outline diagrams (“PM domains”) show the identity of the cell membranes of the dividing cells (#) shown in (D). Grey, red, orange, and green lines represent the basal, apical, lateral, and apicolateral plasma membrane domains, respectively. All figures: filled white arrowheads mark the bile canaliculus or apical domain. Dotted white lines outline the sinusoid (si). Dashed lines indicate the SA. *p<0.05 (calculated using a paired two-tailed Student's *t*-test). Scale bars: 5 µm.

We first analyzed the orientation of the mitotic spindle relative to the apical PA in vivo in mitotic mouse hepatocytes that were in metaphase or in telophase 48 h after hepatectomy. A line drawn through the mitotic spindles poles (immunolabeled with antibodies against the microtubule-binding nuclear mitotic apparatus protein [NuMA]) typically intersected the dipeptidyl peptidase 4 (DPPIV)–positive apical canalicular domains (Figure 2A, arrowheads) or its flanking regions, rather than the basolateral/sinusoidal domains (Figure 2A, sinusoidal domains are indicated by “si” and dotted lines). These data are in agreement with earlier observations in proliferating rat hepatocytes following hepatectomy [27],[28]. Quantification of confocal images of 61 mitotic hepatocytes from three mice 48 h after hepatectomy (see Materials and Methods) revealed that 85.1%±10.1% of the mitotic spindle axes intersected the apical bile canalicular or apicolateral domain (Figure 2B). We also analyzed the orientation of the mitotic SA relative to the apical PA in hepatocytes in vivo in fixed liver tissue from young healthy mice (Figure S1A) and rats (Figure 2C and 2D), which display a burst of cell division after weaning [26],[29]. A line drawn through the NuMA-labeled mitotic spindle poles of rat hepatocytes that were in metaphase or in telophase typically intersected the DPPIV-positive apical bile canalicular plasma membrane domain or its flanking region (Figure 2C). We named this flanking region the apicolateral domain, as it could be distinguished from the “common” lateral plasma membrane facing neighboring cells with which no apical lumen was (yet) formed (Figures 2D and S1B; Movie S1), and is a geometrically distinctive feature of cells with a hepatic polarity phenotype (Figure 1A). The orientation of the mitotic spindle in hepatocytes in vivo correlated well with a restricted localization of the mitotic-spindle-orientating protein LGN [30]–[33] at and/or in close proximity to the zona occludens 1 (ZO-1)–marked tight junctions at this apicolateral region flanking the apical/bile canalicular domain (Figure 2D, green lines in the diagram). LGN was largely absent from the “common” lateral plasma membrane facing neighboring cells with which no apical lumen was shared and absent from basal/sinusoidal plasma membrane domains (Figure 2D, orange and grey lines, respectively, in the diagram). These data demonstrate that in hepatocytes in vivo, the mitotic spindle is predominantly orientated towards an LGN-enriched apical/apicolateral surface domain.

### The Typical Orientation of the Mitotic Spindle to LGN-Enriched Apicolateral Plasma Membrane Domains in Hepatocytes In Vivo Is Reproduced in Cultured HepG2 Cells

**Figure 3:**
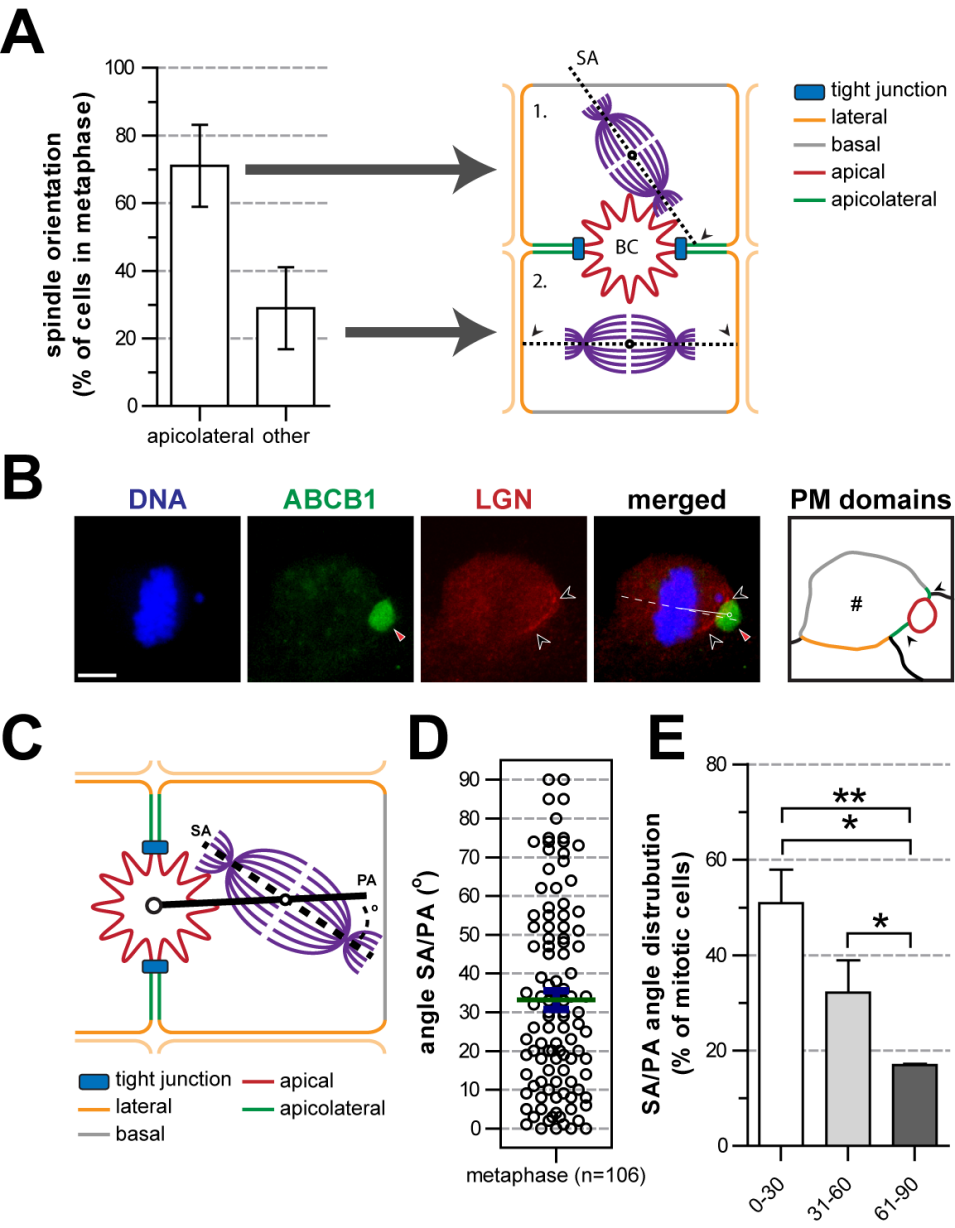
Hepatocytes predominantly orient their mitotic spindle axis towards the apicolateral subdomain. (A) SA axes were quantified as crossing (marked by black arrowheads) the apicolateral membrane (situation 1) or other membranes (situation 2), indicating a bias of the SA axis to cross the apicolateral membrane. (B) Localization of LGN (white outline arrowheads) in polarized HepG2 cells. The apical domain is labeled with ABCB1 and marked by a red arrowhead. The outline diagram (“PM domains”) shows the identity of the cell membranes of the dividing cell (#). Grey, red, orange, and green lines represent the basal, apical, lateral, and apicolateral plasma membrane domains, respectively. (C) Schematic overview of how the orientation of the mitotic spindle (angle between the SA and PA [angle SA/PA]) was measured (see Materials and Methods). (D) Dot plot of the SA/PA angle for dividing HepG2 cells in metaphase. Shown is mean (green bar) and standard error of the mean (SEM) (blue error bars). (E) Histogram analysis reveals a strong bias for HepG2 cells to divide with an SA/PA angle between 0° and 30° during metaphase. *p<0.05; **p<0.01. BC, bile canaliculus. Scale bars: 5 µm.

To investigate the dynamics of mitotic spindle orientation and cell division and its molecular regulation in living hepatocytes, we made use of the polarizing human hepatocyte cell line HepG2 [9],[34]. HepG2 cells develop apical lumens amidst their lateral surfaces facing adjacent cells (Figures 1A and 3), reflecting the earliest stages of apical–basal polarity development in the fetal liver [5],[7],[8],[23],[35]. Importantly, in agreement with the observations in hepatocytes in vivo, a line drawn through the spindle poles in HepG2 cells in more than 70% of all cases intersected the apical or apicolateral domain (Figures 3A and S2A), where LGN was almost exclusively localized (Figure 3B). Note that LGN was not detected at the “common” lateral and sinusoidal plasma membrane domains (Figure 3B, apicolateral domains are green and “common” lateral and sinusoidal domains are orange and grey, respectively, in the diagram). To more accurately determine the orientation of the mitotic spindle relative to the apical PA in these cells, we measured the angle between the SA (the line drawn through the spindle poles; Figure 3C, dotted line) and the PA (the line drawn between the center of the immunolabeled apical domain and the center of the mitotic spindle; Figure 3C, solid line). 50.9%±7.0%, 32.1%±6.8%, and 17.0%±0.3% of the mitotic spindle axes in cells that were in metaphase displayed an SA/PA angle of 0–30°, 31–60°, and 61–90°, respectively, with statistically significant differences between the three categories (Figure 3D and 3E). A similarly biased SA/PA angle distribution was observed in cells that were in later stages of mitosis, i.e., anaphase or telophase (Figure S2A–S2C), and live cell imaging in HepG2 cells showed that the apicolateral-directed orientation of the mitotic spindle was fixed early in mitosis and remained stable throughout the subsequent mitotic stages (Figure S2D and S2E; Movie S2). These data demonstrate that HepG2 cells, similar to hepatocytes in vivo, orient their mitotic spindle with a significant bias towards an LGN-enriched apicolateral plasma membrane domain. These cells are therefore a useful model system to study the consequences of this stereotypic mitotic spindle orientation with regard to cell polarity and its molecular regulation.

### Dividing HepG2 Cells Segregate Their Apical/Bile Canalicular Plasma Membrane Domain Asymmetrically to the Two Emerging Daughter Cells

**Figure 4:**
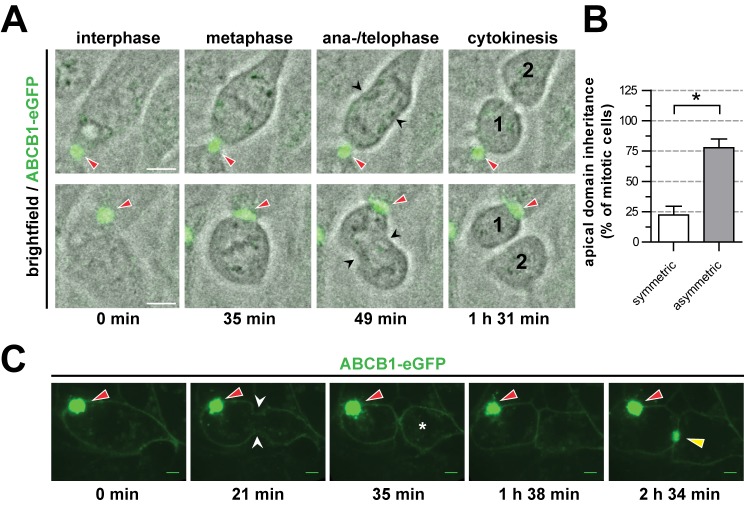
Hepatocytes segregate the apical plasma membrane and lumen asymmetrically during mitosis. (A) Stills from Movie S3 showing asymmetric segregation of the apical plasma membrane (ABCB1-eGFP, red arrowheads) in dividing HepG2 cells. Black arrowheads mark the ingressing cleavage furrow during cytokinesis. “1” marks the daughter cell inheriting the apical domain, and “2” marks the daughter cell not inheriting the apical domain, hence becoming non-polarized. (B) Quantification of the asymmetry of apical plasma membrane inheritance in dividing HepG2 cells (live imaging; *n* = 64). (C) Stills from Movie S5 showing asymmetric segregation of the apical domain (ABCB1-eGFP, red arrowheads) and formation of a new apical domain by the new daughter cell. White arrowheads mark the ingressing cleavage furrow. The yellow arrowhead marks the de novo formed apical domain at the site of cytokinesis. *p<0.05. Scale bars: 5 µm.

Concomitant with the predominant apicolateral-plasma-membrane-directed orientation of the mitotic spindle apparatus, live cell imaging revealed that HepG2 cells predominantly divided in such a way that only one of the two emerging daughter cells inherited the apical lumen. Stills from a representative movie (Movie S3) of dividing HepG2 cells that express the green fluorescent eGFP-tagged apical protein ABCB1 (Figure 4A) show two non-mitotic (interphase) cells with ABCB1-eGFP-positive apical plasma membrane domains and lumens (indicated by the red arrowhead). After each cell passed through metaphase it formed a cleavage furrow (Figure 4A, black arrowheads) during anaphase/telophase that, following subsequent cytokinesis, gave rise to one daughter cell (marked by “1”) that inherited the apical domain (red arrowhead) and one daughter cell (marked by “2”) that did not. The vast majority of live cells (>75%) showed this asymmetric segregation of the apical plasma membrane domain and lumen during mitosis (Figure 4B). Similar results were observed with another hepatocyte cell line, WIF-B9 (Figure S3A and S3B; Movie S4), underscoring that this mode of cell division orientation is a feature of polarized hepatocytes and not only of HepG2 cells. Interestingly, we observed that the emerging non-polarized daughter cells could reestablish apical–basal polarity and reestablish an apical lumen with their new neighbor cells. An example of this is shown in the stills (Figure 4C) from Movie S5. These stills show that a HepG2 cell with an ABCB1-eGFP-positive apical plasma membrane domain and lumen (red arrowhead) formed a cleavage furrow (white arrowheads) that gave rise to one daughter cell (marked by the asterisk) that did not inherit the apical domain (red arrowhead), but reestablished an ABCB1-eGFP-positive apical domain (yellow arrowhead) with its sister at the site of cytokinesis. Taken together, we conclude that hepatocytes orientate their mitotic SA with a significant bias towards an LGN-enriched apicolateral plasma membrane domain, and asymmetrically segregate their apical plasma membrane domain and apical lumen to the emerging daughter cells.

### Par1b Overexpression in Simple Epithelial Cells Coordinates the Adoption of a Hepatic Polarity Phenotype with Changes in the Localization of LGN and Mitotic Spindle and Cell Division Orientation

**Figure 5:**
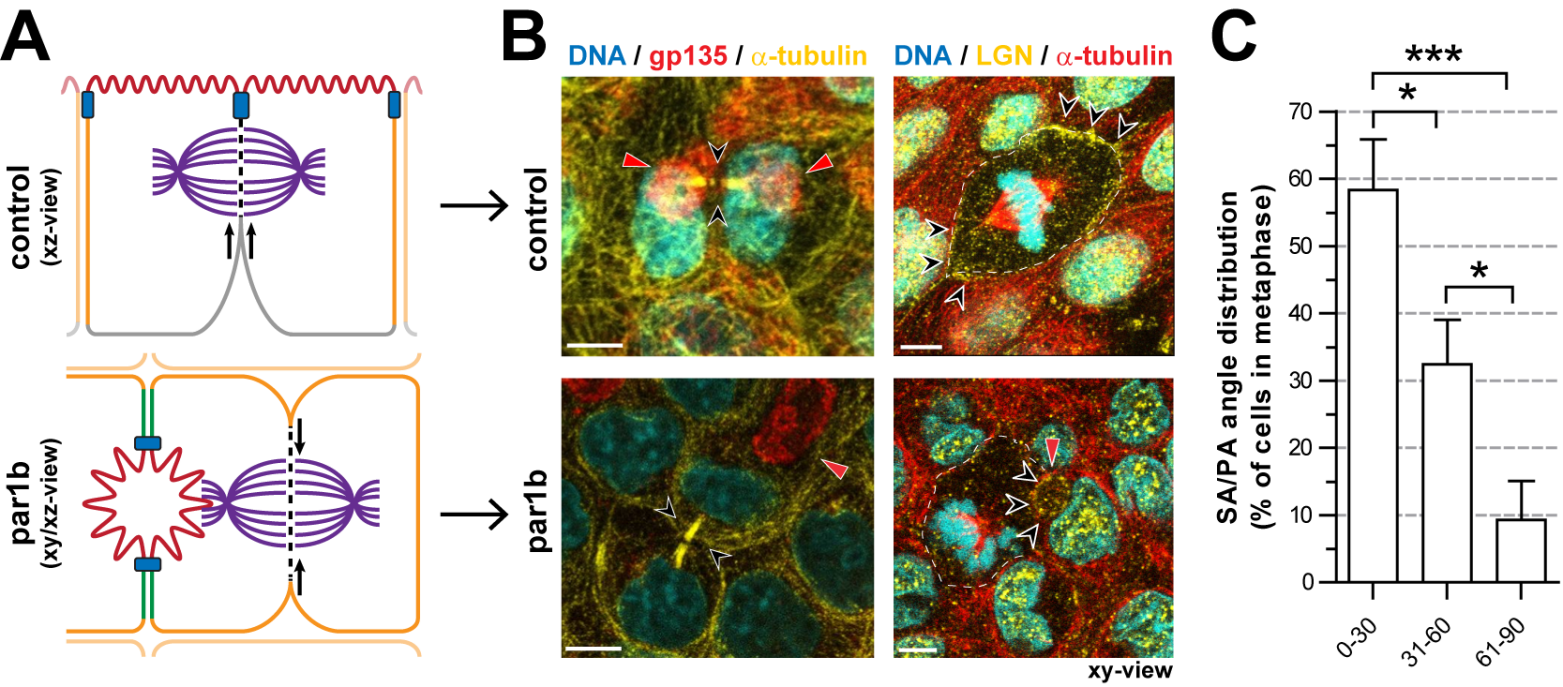
Par1b stimulates apicolateral-directed spindle orientation and asymmetric segregation of the apical domain in MDCK cells. (A) Schematic overview of the polarity phenotype in control and Par1b-overexpressing MDCK cells. (B) Fixed control and MDCK-Par1b cells were labeled for the apical marker gp135 (left panel, red) and LGN (right panel, yellow). Par1b-overexpressing MDCK cells asymmetrically segregate their apical domain (left panel, red arrowheads; black arrowheads mark the ingressing cleavage furrow) during cell division. LGN localizes to the apicolateral plasma membrane domain in Par1b-overexpressing MDCK cells (right panel, black arrowheads). The dashed line marks the common lateral plasma membrane domain. (C) Histogram analysis of the SA/PA angle shows that dividing MDCK-Par1b cells exhibit a bias towards lower angles (0–30°) in metaphase, as observed for hepatocytes. All figures: red arrowheads mark the apical domain. *p<0.05. ***p<0.001. Scale bars: 5 µm.

In order to investigate to what extent the hepatocyte polarity phenotype—as such—was sufficient to dictate an apicolateral-domain-directed orientation of the mitotic spindle, we made use of our earlier observation that the overexpression of the polarity protein partitioning-defective 1/microtubule-affinity regulating kinase 2 (Par1b/MARK2) in simple epithelial cells induces a hepatic polarity phenotype (schematically depicted in Figure 5A) [22]. Thus, when Par1b was overexpressed in MDCK cells (a widely used model of simple epithelial cells), apical plasma membrane proteins such as gp135 localized to lumens formed between adjacent cells (Figure 5B, Par1b), rather than to the cell-culture-medium-facing cell surface at the top of the control cell monolayer (Figure 5B, control). In mitotic cells identified in parental MDCK cell cultures, LGN was localized at the lateral plasma membrane domains and was excluded from the apical domain (Figure 5B, control, dotted white line; and previous reports [19]). In agreement with the role of cortical LGN in spindle orientation in these cells [19], 100% of all SA/PA angles have been demonstrated to be in the 61–90° range, giving rise to the symmetric segregation of apical and basal domains to both daughter cells [36],[37]. In contrast, in MDCK cells that overexpressed Par1b and displayed a hepatic polarity phenotype, the localization of LGN was highly polarized and restricted to the apical/apicolateral domain (Figure 5B, Par1b, red arrowhead), and LGN was excluded from the “common” lateral cell surfaces (Figure 5B, Par1b, dotted white line). Coinciding with this change in the distribution of LGN, 58.3%±7.5%, 32.4%±6.6%, and only 9.3%±5.8% of all SA/PA angles were in the 0–30°, 31–60°, and 61–90° range, respectively, with a clear, statistically significant difference between these categories (Figure 5C). These data demonstrate that the overexpression of Par1b in simple epithelial cells caused a strong shift from a mitotic spindle orientation that was perpendicular to the apical PA to one that was significantly more parallel to the apical PA, matching a change in the distribution of LGN. Concomitantly, live cell imaging revealed that Par1b-overexpressing MDCK cells, like hepatic cells, predominantly divided in such a way that only one of the two emerging daughter cells inherited the apical lumen (Figure S4A–S4C; Movie S6). These data show that Par1b coordinates the induction of a hepatic polarity phenotype with a change in (1) the localization of LGN, (2) the orientation of the mitotic spindle, and (3) the asymmetric segregation of the apical plasma membrane domain to the emerging daughter cells.

### Par1b Regulates Mitotic Spindle Orientation by Controlling the Apicolateral Enrichment of LGN in HepG2 Cells

**Figure 6:**
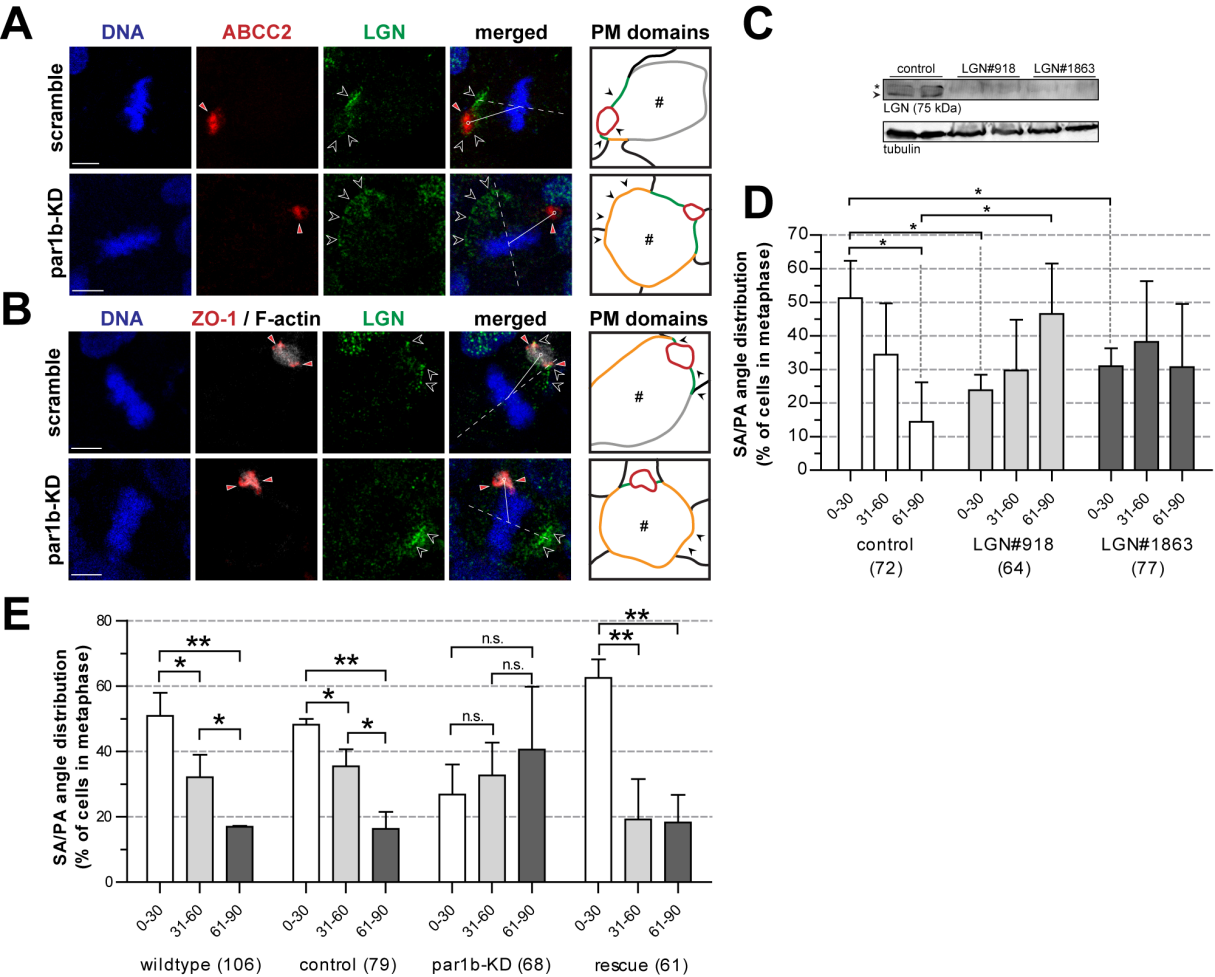
LGN accumulates at the apicolateral subdomain in a Par1b-dependent manner and controls spindle orientation. (A and B) Localization of LGN (white outline arrowheads) in control (scrambled) and Par1b shRNA HepG2 cells. The apical domain (red arrowheads) is labeled with ABCC2 (A) or F-actin (B). Tight junctions are labeled with ZO-1 (B). The dashed line represents the SA. The solid line represents the PA. The outline diagrams (“PM domains”) show the identity of the cell membranes of the dividing cells (#). Grey, red, orange, and green lines represent the basal, apical, lateral, and apicolateral plasma membrane domains, respectively. (C) Western blot analysis of LGN knockdown in HepG2 cells using two shRNA constructs. (D) Histogram analysis of SA/PA angles in LGN knockdown HepG2 cells indicating a loss of bias towards lower angles (0–30°) under LGN knockdown conditions. (E) Histogram analysis of wild type, control (scrambled) shRNA, Par1b knockdown, and Par1b rescued HepG2 cells shows a loss of apicolateral-directed spindle orientation under Par1b knockdown conditions. *p<0.05. **p<0.01. n.s., not significant. Scale bars: 5 µm.

The results from the Par1b-overexpressing MDCK cells as displayed in Figure 5 do not demonstrate whether solely the induction of a hepatic polarity phenotype—as such—was sufficient to change the localization of LGN and the orientation of the mitotic spindle. Therefore, in order to further investigate to what extent Par1b is important for the localization of LGN and spindle orientation in the context of the hepatic polarity phenotype, we knocked down Par1b in HepG2 cells (Figure S5A). Confocal images in Figure 6A and 6B show that in control cells—i.e., cells treated with scrambled shRNA (scramble)—LGN (black arrowheads) localized at the cell surface flanking the ABCC2- or ZO-1/actin-labeled apical domain (red arrowheads), similarly to in untreated cells (cf. Figure 3B). In contrast, in cells treated with shRNA against Par1b (Par1b-KD), the localization of LGN was no longer restricted to the apicolateral domain (Figure 6A and 6B). LGN frequently showed an (additional) localization at the “common” lateral domain (black arrowheads, the “common” lateral domain is orange in the diagram), away from the apical domain (Figure 6A and 6B, red arrowhead). Note that these cells have retained the typical hepatic polarity phenotype. The change in the distribution profile of LGN was accompanied by a change in the orientation of NuMA-positive astral microtubules that emanated from the mitotic spindle poles and reached out to the cell cortex (*z*-stack sections in Figure S5B; Movies S7 and S8). The knockdown of LGN in HepG2 cells with two different shRNAs (Figures 6C and S6A) caused a randomization of the SA/PA angle in the 0–30°, 31–60°, and 61–90° categories, and caused a significant reduction of SA/PA angles in the 0–30° category when compared to control cells (Figures 6D, S6B, and S6C), hence underscoring the contribution of LGN to mitotic spindle orientation in these cells. In agreement with the change in LGN distribution, the knockdown of Par1b caused a randomization of the orientation of the mitotic spindle axes, with approximately 26.8%±9.2%, 32.6%±10.1%, and 40.6%±19.2% displaying an SA/PA angle between 0–30°, 31–60°, and 61–90°, respectively, with no statistically significant difference between the three categories (Figure 6E). Thus, Par1b knockdown effectively abolished the bias in mitotic SA orientation towards smaller SA/PA angles (0–30°). An illustrative example of this is shown in Figures 6A, 6B, and S5D, and the quantifications are depicted in Figures 6E and S5C. The reintroduction of shRNA-resistant Par1b (Figure S5A) completely rescued this effect, and treatment of the cells with a scrambled shRNA without effect on Par1b expression did not affect the SA/PA distributions (Figure 6E, control and rescue). Similarly to in HepG2 cells, the knockdown of Par1b in WIF-B9 cells, or the expression of a dominant-negative Par1b mutant, caused a statistically significant shift in SA/PA angle bias from 0–30° to 31–60° and 61–90° (Figure S7A–S7C), underscoring that the role of Par1b mitotic spindle orientation is a feature of polarized hepatocytes and not only of HepG2 cells. Concomitant with the loss of bias towards 0–30° angles, live cell imaging showed that the frequency of cell divisions in which both daughter cells inherited part of the same apical lumen significantly increased upon Par1b depletion or expression of the Par1b-DN mutant (Figure S7D–S7F; Movie S9). Taken all together, our data suggest that, in cells with a hepatic type polarity, Par1b controls the apicolateral enrichment of LGN and, thereby, the apicolateral-directed orientation of the mitotic spindle to promote the asymmetric segregation of the apical plasma membrane domain to the two emerging daughter cells and to preserve the typical polarity phenotype of polarized hepatocytes.

## Discussion

This study demonstrates that mitotic hepatocytes asymmetrically segregate their apical plasma membrane domains to the emerging daughter cells during cell division. This is in striking contrast to the symmetric segregation of apical and basal surface domains observed in vitro and in vivo in simple epithelial cells such as those found in the neuroepithelium, kidney, and intestine (reviewed in [15]).

**Figure 7:**
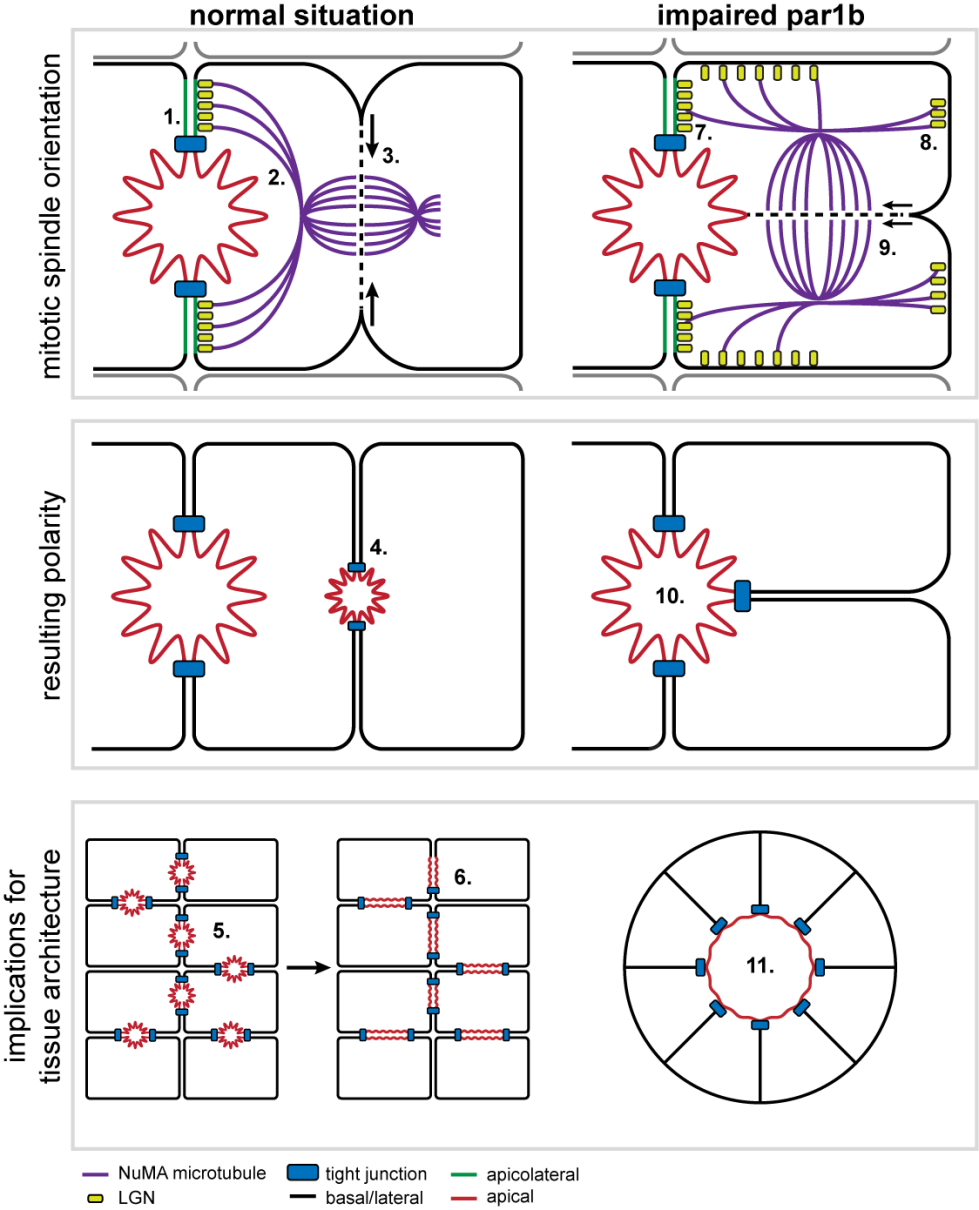
Implications of mitotic spindle orientation during the development of the unique liver architecture. (1) LGN localizes to the apicolateral plasma membrane area during hepatocyte cell division. (2) The mitotic spindle orients one of its (NuMA-containing) spindle poles towards the LGN-enriched apicolateral plasma membrane. (3) This orientation of the mitotic spindle results in the cleavage furrow not bisecting the apical plasma membrane, resulting in asymmetric segregation of the apical plasma membrane. (4) New apical surfaces are created de novo at the site of abscission. (5) During early liver development, apical pockets are created between hepatocytes. (6) These pockets grow out to bile canalicular/channel-like structures during later phases of liver development. (7) When Par1b is impaired, LGN migrates away from the apicolateral plasma membrane area and is subsequently found on basal or lateral membranes. (8) The mitotic spindle orients its poles towards LGN-enriched cortical areas. (9) The cleavage furrow has an increased chance of bisecting the apical plasma membrane, resulting in symmetric segregation of the apical plasma membrane. (10) Both cells now share the same apical surface (“simple” epithelial polarity). (11) Continued cell division likely results in the generation of “simple” epithelial cyst-like structures.

Our data indicate that this asymmetric inheritance of the apical plasma membrane domain in hepatocytes is dictated by an apicolateral-plasma-membrane-domain-directed orientation of the mitotic spindle. Interestingly, this apicolateral plasma membrane domain is a geometrically distinctive feature in cells with a (fetal) hepatic polarity phenotype (Figure 1A, apicolateral domain is green in the diagram). Indeed, the apicolateral domain represents the cell's contacting surface with the adjoining cell with which it shares an apical lumen, and can be distinguished from its contacting surface with other adjoining cells with which no apical lumens are (yet) shared (Figure 7, orange). This apicolateral subdomain has gone unnoticed, presumably because no functional relevance had been ascribed to it. Our data now demonstrate that Leu-Gly-Asn repeat-enriched protein (LGN) predominantly accumulates at this apicolateral domain during mitosis, both in rat liver hepatocytes in vivo and in polarized HepG2 cells in culture. Furthermore, NuMA-positive astral microtubules predominantly target this apicolateral domain in mitotic HepG2 cells. These observations are consistent with data from other cell systems in which LGN recruits NuMA on astral microtubules to the cell cortex (reviewed in [13],[15],[38]). Indeed, knockdown experiments demonstrate that LGN is necessary for orientating the SA predominantly towards the apicolateral domain. We propose that this apicolateral domain thus serves as an instructive positional landmark in hepatocytes for the polarized recruitment of LGN, which, in turn, is required for the predominantly apicolateral orientation of the mitotic spindle and asymmetric segregation of the apical domain to the nascent daughter cells.

In contrast to the apicolateral accumulation of LGN in dividing hepatocytes, LGN has been shown to accumulate at the apical plasma membrane domain in asymmetrically dividing Drosophila neuroblasts [39],[40]. In epithelial cells, atypical protein kinase C at the apical plasma membrane has been proposed to exclude the apical recruitment of LGN [41],[42]. Possibly, the presence of atypical protein kinase C at the apical bile canalicular plasma membrane domain in HepG2 cells and primary hepatocytes (unpublished data) may have similarly prevented the accumulation of LGN at the apical surface. But what caused LGN accumulation at the apicolateral subdomain and excluded it from the “common” lateral surface in hepatocytes? Our data implicate the polarity protein Par1b as a critical determinant for this. Indeed, upon knockdown of Par1b in HepG2 cells, LGN no longer accumulated predominantly at the apicolateral domain, but rather showed additional localization at “common” lateral plasma membrane domains. In agreement with the occurrence of multiple sites of cortical LGN, NuMA-positive astral microtubules reached multiple sites at the cell surface. Concomitant with the altered distribution of LGN, knockdown of Par1b or expression of a nonfunctional Par1b mutant in HepG2 and WIF-B9 cells resulted in a loss of spindle orientation bias towards the apicolateral domain and promoted symmetric cell divisions that bisected the apical surface. Notably, in Par1b-depleted hepatic cells the loss of apicolaterally restricted LGN occurred while cells maintained their apicolateral domain. This suggests that Par1b translated the presence of the apicolateral domain as a positional landmark to cortical polarity—i.e., the apicolateral accumulation of LGN—in the mitotic cell. This is further supported by our observations that the overexpression of Par1b in simple epithelial MDCK cells coordinated the acquisition of a fetal hepatocyte polarity phenotype (and thus the establishment of an apicolateral domain) with apicolateral LGN recruitment during mitosis, spindle orientation, and asymmetric cell division. This demonstrates that the coordinated action of Par1b and LGN constitutes a fundamental part of the molecular mechanism that drives mitotic spindle orientation and asymmetric/symmetric apical plasma membrane inheritance. Further studies are needed to determine how Par1b precisely controls the exclusive apicolateral recruitment of LGN.

The orientation of cell division parallel to the apical–basal axis establishes cell fate specification, as has been shown in skin epithelial cells [43],[44] and in neuroblasts [15], although not necessarily [31]. Apart from the asymmetric inheritance of apical plasma membrane proteins, we observed no overt signs of cell fate specification in dividing HepG2 cells. Live cell imaging showed that emerging daughter cells that did not inherit the apical plasma membrane domain were capable of establishing an apical plasma membrane domain and lumen with a new neighbor or the sister cell, and did not show distinct behavior when compared to the polarized daughter cell. We cannot, however, exclude the asymmetric acquisition of specific molecules that may have endowed one of the daughter cells with distinct capabilities. Although fetal liver development/patterning has not been experimentally tested, our findings may suggest that in early fetal hepatocytes the asymmetric segregation of apical domains during division may, in conjunction with a repolarization of non-polarized nascent daughter cells, promote the dissemination of isolated apical lumens throughout the proliferating cell mass. It can be speculated that, in vivo, such asymmetric-cell-division-driven propagation of biliary luminal pockets throughout the proliferative fetal liver parenchymal mass could facilitate the development of a branching canalicular network via concomitant or subsequent apical lumen expansion and fusion (Figure 7). Indeed, elongation of bile canaliculi results from the expansion and fusions of numerous small isolated apical lumens [1],[2],[5] and as such would not necessarily require symmetric cell divisions. We propose that the Par1b-regulated spindle orientation via LGN and the resultant asymmetric inheritance of individual apical plasma membrane domains and lumens, as shown in this study, are made possible by and serve the unique polarized architecture of hepatocytes and, possibly, the liver parenchymal tissue.

To our knowledge, there have been no reports in the literature on human liver diseases associated with Par1b, LGN, or hepatic spindle disorientation. Defects in the orientation of the mitotic spindle apparatus may hamper the efficient development of bile canalicular networks during normal liver development or regeneration, or promote the development of cystic lumens, the latter process typically being driven by symmetric divisions [17]–[19]. In future studies Par1b knockout mice may be useful to investigate the role of Par1b in the formation of the bile canalicular network during embryonic liver development or regeneration after hepatectomy.

## Supporting Information

Figure S1 **Mouse and rat hepatocytes orient their mitotic spindle axis towards the apicolateral subdomain.** (A) Shown is a near-native tissue slice (100 µm) of weaned mouse liver stained for DNA, bile canaliculus (DPPIV/CD26), and the sinusoid (mouse, CD31). The SA intersects the apicolateral domain. Dotted white lines outline the sinusoid (si). (B) Immunofluorescence labeling of the bile canalicular protein DPPIV (green) and the cell–cell adhesion junction–associated protein beta-catenin in 2-d postnatal rat hepatocytes. Apicolateral and “common” lateral plasma membrane domains, color-coded in the diagram, can be distinguished. The # marks the cell for which the membranes were distinguished. See also Movie S1. Scale bars: 5 µm.
(TIF)

Figure S2 **Hepatocytes predominantly orient their mitotic spindle axis towards the apicolateral subdomain and asymmetrically segregate their apical plasma membrane.** (A) Illustration of HepG2 cells in various mitotic phases for which the SA/PA angle was calculated (the asterisk marks the apical domain to which the SA/PA angle was calculated). The apical domain is labeled with ABCB1. The microtubules of the mitotic spindle were labeled with β-tubulin. (B) Dot plot of SA/PA angles for dividing HepG2 cells for the various phases shown in (A). Shown is mean (green bar) and SEM (blue error bars). (C) Histogram analysis reveals a strong bias for HepG2 cells to divide with an SA/PA angle between 0° and 30° during metaphase, anaphase, and telophase. (D and E) A closer examination of the real-time dynamics of spindle orientation during mitosis by live cell imaging (D) (stills from Movie S2; DNA labeled by H2B-mCherry, the apical domain labeled by ABCB1-eGFP and red arrowheads; black arrowheads mark the ingressing cleavage furrow) reveals that the SA/PA angle oscillates between −15° and 15° relative to the apical–basal axis (E) (blue line; cell from Movie S2), while maintaining the same spindle pole facing the apicolateral domain. Prior to the onset of anaphase, the SA appears stabilized at a fixed orientation and shows minimal if any rotation during the subsequent course of mitosis (E) (green and orange lines; Movie S2). *p<0.05. **p<0.01. Scale bars: 10 µm (A) and 5 µm (D).
(TIF)

Figure S3 **WIF-B9 cells segregate the apical plasma membrane and lumen asymmetrically during mitosis.** (A) Stills from Movie S4. WIF-B9 cells, labeled with DRAQ5 to label chromatin/DNA, showing asymmetric and symmetric segregation of the apical plasma membrane (red arrowheads). (B) The graph represents a quantification of the asymmetry of apical domain inheritance in dividing WIF-B9 cells (live imaging; *n* = 27). **p<0.01. Scale bars: 10 µm.
(TIF)

Figure S4 **Asymmetric segregation of the apical plasma membrane domain in Par1b-overexpressing MDCK cells.** (A) Dot plot of SA/PA angles for dividing MDCK-Par1b cells. Shown is mean (green bar) and SEM (blue error bars). (B and C) Time-lapse analysis (stills from Movie S6) (B) and quantification (C) of control and MDCK-Par1b cells, indicating symmetric and asymmetric inheritance of apical plasma membrane domains in control and MDCK-Par1b cells, respectively. ***p<0.001. Scale bars: 5 µm.
(TIF)

Figure S5 **Par1b regulates apicolateral-directed spindle orientation in HepG2 cells.** (A) Quantification of Par1b knockdown in HepG2 cells by quantitative PCR. (B) Stills from Movies S7 and S8, showing cortical NuMA (black arrowheads) at the apical domain and at both the apical and lateral membranes in control and Par1b knockdown HepG2 cells, respectively. (C) SA/PA angle was calculated for control (scrambled) shRNA and Par1b knockdown HepG2 cells (fixed) as indicated in (D) and plotted as depicted. All figures: red arrowheads mark the apical domain. The outlines show the identity of the cell membranes of the dividing cells (#) shown in (A). Red, orange, and green lines represent the apical, lateral, and apicolateral plasma membrane domains, respectively. **p<0.01. Scale bars: 5 µm.
(TIF)

Figure S6 **Short hairpin RNA targeted against LGN results in the depletion of the LGN protein in HepG2 cells, which, in turn, perturbs the apicolateral orientation of the mitotic spindle apparatus.** (A) Real-time PCR analysis of the knockdown efficiency of the two LGN constructs used in this study. (B) Dot plot of SA/PA angles of HepG2 cells in metaphase under control and LGN knockdown conditions. Shown is mean with SEM. (C) Shown is the percentage of HepG2 cells with SA crossing the apicolateral membrane under control and LGN knockdown conditions, indicating reduced apicolateral spindle orientation under LGN knockdown conditions. *p<0.05. **p<0.01.
(TIF)

Figure S7 **Knockdown of Par1b in WIF-B9 cells alters the orientation of the mitotic spindle apparatus relative to the apical polarity axis.** (A) Dot blot showing individual SA/PA angles for dividing WIF-B9 cells under the depicted conditions. Shown is mean with SEM. (B) Histogram analysis of control (pSUPER) and Par1b-KD WIF-B9 cells indicating reduced apicolateral-subdomain-oriented spindle orientation (reduced bias towards lower angles [0–30°]) during Par1b depletion. (C) Histogram analysis of control (GFP) and Par1b-DN-GFP-expressing WIF-B9 cells indicating reduced apicolateral-subdomain-oriented spindle orientation (reduced bias towards lower angles [0–30°]) when Par1b function is perturbed. (D) WIF-B9 cells expressing Par1b-DN and Par1b knockdown cells in metaphase were scored for symmetric or asymmetric segregation of the apical plasma membrane. Reduced Par1b activity increased symmetric inheritance of the apical plasma membrane. (E) Illustrations of dividing control and Par1b-DN-expressing WIF-B9 cells. DPPIV marks the apical domain, β-tubulin marks the microtubules of the mitotic spindle. (F) Symmetry of cell division was also quantified during live imaging of WIF-B9 cells. Black arrowheads mark the ingressing cleavage furrow (midbody, site of cytokinesis). *p<0.05. **p<0.01. ***p<0.001. Scale bars: 5 µm.
(TIF)

Movie S1 **Distinct apicolateral and “common” lateral surface domains can be distinguished in postnatal rat hepatocytes.** *z*-stack (*z*-step 1.0 µm, 15 *z*-sections; 5 frames per second [fps]) of a rat liver hepatocyte in a postnatal day 2 liver showing beta-catenin (red), DPPIV (green), and nuclei (blue).
(MOV)

Movie S2 **Dynamics of mitotic spindle orientation in HepG2 cells.** HepG2 cells stably expressing ABCB1-eGFP were transfected with H2B-mCherry and grown and live-imaged (4 min/frame; 7 fps) as described for Movie S1. Shown is a cell that asymmetrically segregates its apical plasma membrane without significant spindle rotation, indicating that the orientation of the mitotic spindle is fixed in early metaphase.
(MOV)

Movie S3 **HepG2 cells asymmetrically segregate their apical plasma membrane domain and lumen to the emerging daughter cells.** HepG2 cells stably expressing ABCB1-eGFP were grown for 2 d and live-imaged on a spinning disk confocal microscope (7 min/frame; 30 fps). Black arrowheads depict polarized HepG2 cells segregating their apical plasma membrane—marked by ABCB1-eGFP—asymmetrically.
(MOV)

Movie S4 **Orientation of cell division in polarized WIF-B9 cells.** WIF-B9 cells, labeled with DRAQ5 to label chromatin/DNA, were followed with time-lapse confocal microscopy (15 min/frame; 7 fps) and show asymmetric and symmetric segregation of the apical plasma membrane (red arrowheads mark the bile canaliculus). Scale bars: 10 µm.
(MOV)

Movie S5 **Emerging non-polarized HepG2 daughter cells reestablish apical polarity following cytokinesis.** HepG2 cells stably expressing ABCB1-eGFP were grown and live-imaged (7 min/frame; 30 fps) as described for Movie S3. The new daughter cell is able to make a new apical domain with the mother cell. Scale bar: 5 µm.
(MOV)

Movie S6 **Symmetry of cell division in MDCK and Par1b-overexpressing MDCK cells.** Control and Par1b-expressing MDCK cells were labeled with DRAQ5 and transfected with gp135-GFP to mark the apical plasma membrane, followed by time-lapse confocal microscopy (10 min/frame; 7 fps). Control MDCK cells segregate their apical domain symmetrically, while Par1b-expressing MDCK cells segregate the apical plasma membrane asymmetrically, as indicated.
(MOV)

Movie S7 **NuMA-positive astral microtubules predominantly reach the apicolateral domain in HepG2 cells.** *z*-stack (*z*-step 0.25 µm; 5 fps) of a polarized dividing HepG2 cell showing NuMA-positive astral microtubules (green) traveling towards the apical plasma membrane (red).
(MOV)

Movie S8 **Knockdown of Par1b in HepG2 cells alters the distribution of NuMA-positive astral microtubules.** *z*-stack (*z*-step 0.25 µm; 5 fps) of a polarized dividing Par1b knockdown HepG2 cell showing NuMA-positive astral microtubules (green) traveling towards the apical plasma membrane (red) and lateral membranes.
(MOV)

Movie S9 **Symmetric segregation of apical plasma membrane in WIF-B9 cells.** Time-lapse experiment (10 min/frame; 10 fps) showing a WIF-B9 cell segregating its apical plasma membrane symmetrically.
(MOV)

Table S1 **A list of the commercial antibodies used in this study.**
(DOCX)

Table S2 **RNA interference target sequences for HepG2 cells.** Listed are the sense sequences used to generate oligonucleotides according to the pLKO manual.
(DOCX)

Table S3 **Real-time PCR primers used in this study.** Listed are forward and reverse primer DNA sequences.
(DOCX)

## Abbreviations

fps: frames per second
MDCK: Madin-Darby canine kidney
PA: polarity axis
SA: spindle axis
SEM: standard error of the mean
shRNA: short hairpin RNA
