# Correction: Par1b Induces Asymmetric Inheritance of Plasma Membrane Domains via LGN-Dependent Mitotic Spindle Orientation in Proliferating Hepatocytes

**DOI:** 10.1371/annotation/aacd66b6-d706-4f59-a79a-5d55f83d17ca

**Published:** 2014-01-13

**Authors:** Christiaan L. Slim, Francisco Lázaro-Diéguez, Marjolein Bijlard, Mathilda J. M. Toussaint, Alain de Bruin, Quansheng Du, Anne Müsch, Sven C. D. van IJzendoorn

There were errors in the Funding section. The correct funding information is as follows: FL-D and AM were funded by National Institute of Health grant R01KD064842-07A1. QD was funded by National Institute of Health grant GM079506. The funders had no role in study design, data collection and analysis, decision to publish, or preparation of the manuscript.

