# Supplementary figures and images for: Par1b Induces Asymmetric Inheritance of Plasma Membrane Domains via LGN-Dependent Mitotic Spindle Orientation in Proliferating Hepatocytes
Source: PLoS Biol. 2013 Dec 17;11(12):e1001739. doi: 10.1371/journal.pbio.1001739 (PMC3866089; doi:10.1371/journal.pbio.1001739)

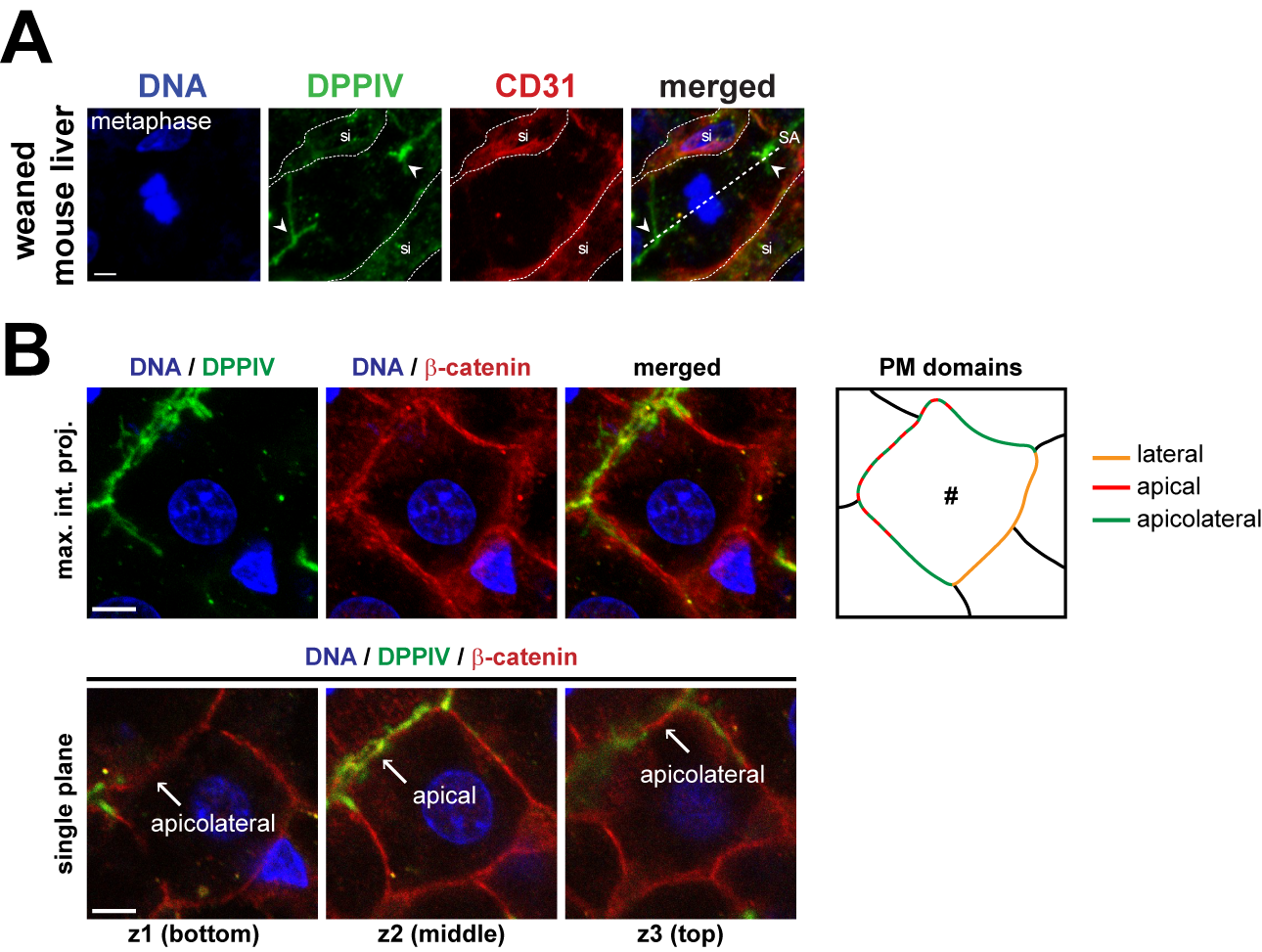

Supplement: Figure S1 — Mouse and rat hepatocytes orient their mitotic spindle axis towards the apicolateral subdomain. (A) Shown is a near-native tissue slice (100 µm) of weaned mouse liver stained for DNA, bile canaliculus (DPPIV/CD26), and the sinusoid (mouse, CD31). The SA intersects the apicolateral domain. Dotted white lines outline the sinusoid (si). (B) Immunofluorescence labeling of the bile canalicular protein DPPIV (green) and the cell–cell adhesion junction–associated protein beta-catenin in 2-d postnatal rat hepatocytes. Apicolateral and “common” lateral plasma membrane domains, color-coded in the diagram, can be distinguished. The # marks the cell for which the membranes were distinguished. See also Movie S1. Scale bars: 5 µm. (TIF) [file pbio.1001739.s001.tif]

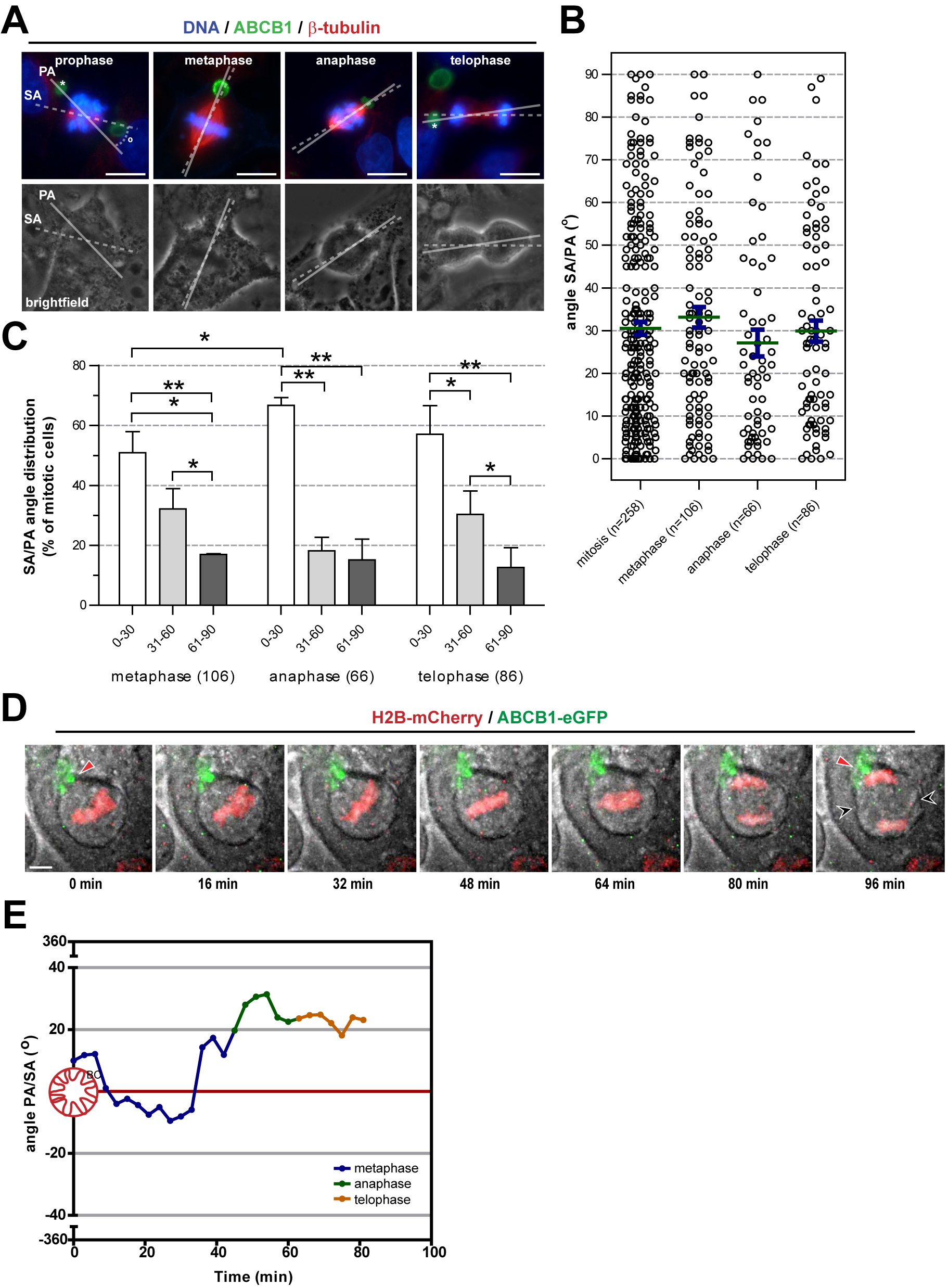

Supplement: Figure S2 — Hepatocytes predominantly orient their mitotic spindle axis towards the apicolateral subdomain and asymmetrically segregate their apical plasma membrane. (A) Illustration of HepG2 cells in various mitotic phases for which the SA/PA angle was calculated (the asterisk marks the apical domain to which the SA/PA angle was calculated). The apical domain is labeled with ABCB1. The microtubules of the mitotic spindle were labeled with β-tubulin. (B) Dot plot of SA/PA angles for dividing HepG2 cells for the various phases shown in (A). Shown is mean (green bar) and SEM (blue error bars). (C) Histogram analysis reveals a strong bias for HepG2 cells to divide with an SA/PA angle between 0° and 30° during metaphase, anaphase, and telophase. (D and E) A closer examination of the real-time dynamics of spindle orientation during mitosis by live cell imaging (D) (stills from Movie S2; DNA labeled by H2B-mCherry, the apical domain labeled by ABCB1-eGFP and red arrowheads; black arrowheads mark the ingressing cleavage furrow) reveals that the SA/PA angle oscillates between −15° and 15° relative to the apical–basal axis (E) (blue line; cell from Movie S2), while maintaining the same spindle pole facing the apicolateral domain. Prior to the onset of anaphase, the SA appears stabilized at a fixed orientation and shows minimal if any rotation during the subsequent course of mitosis (E) (green and orange lines; Movie S2). *p<0.05. **p<0.01. Scale bars: 10 µm (A) and 5 µm (D). (TIF) [file pbio.1001739.s002.tif]

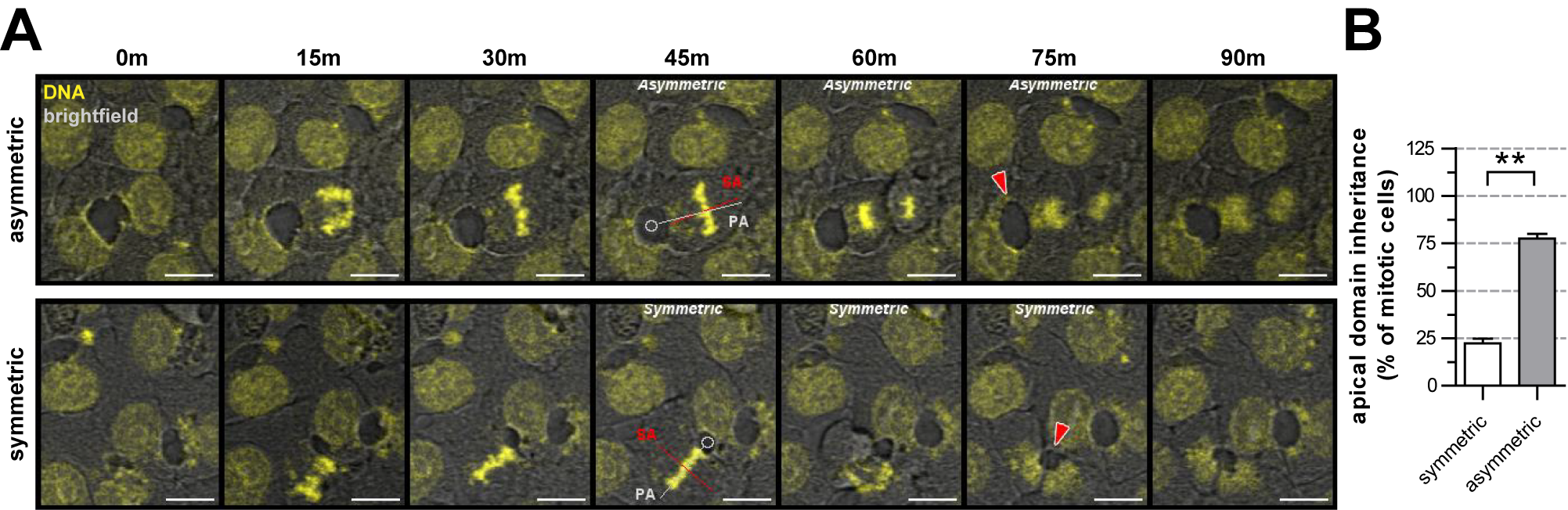

Supplement: Figure S3 — WIF-B9 cells segregate the apical plasma membrane and lumen asymmetrically during mitosis. (A) Stills from Movie S4. WIF-B9 cells, labeled with DRAQ5 to label chromatin/DNA, showing asymmetric and symmetric segregation of the apical plasma membrane (red arrowheads). (B) The graph represents a quantification of the asymmetry of apical domain inheritance in dividing WIF-B9 cells (live imaging; n = 27). **p<0.01. Scale bars: 10 µm. (TIF) [file pbio.1001739.s003.tif]

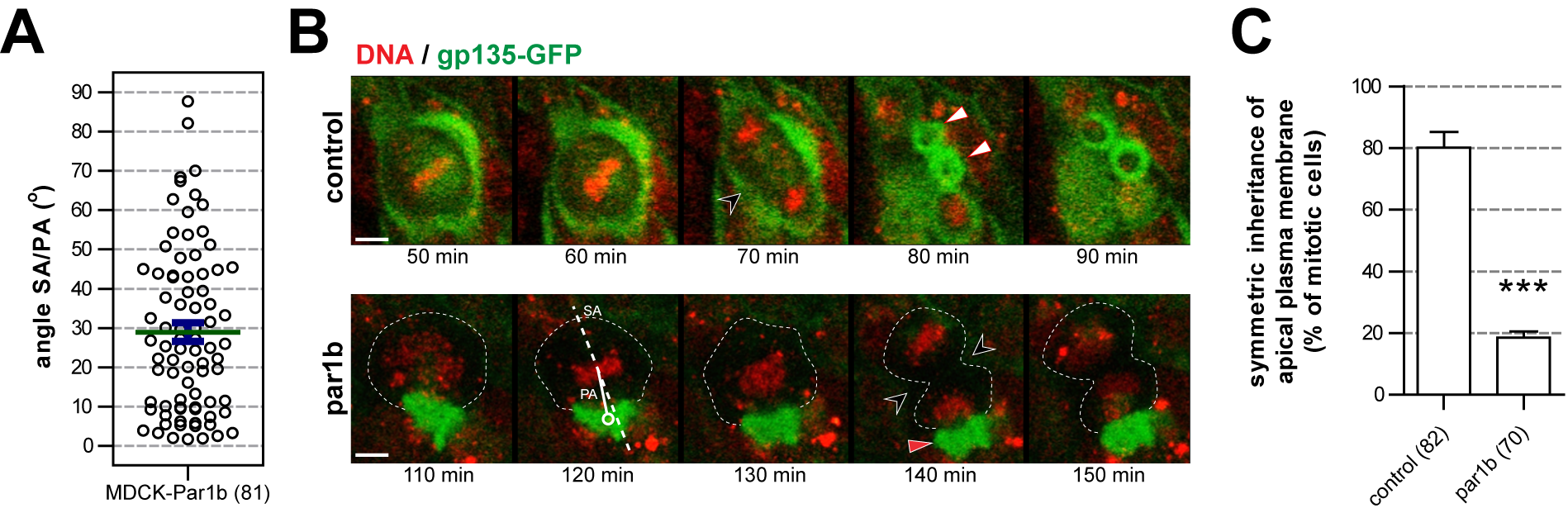

Supplement: Figure S4 — Asymmetric segregation of the apical plasma membrane domain in Par1b-overexpressing MDCK cells. (A) Dot plot of SA/PA angles for dividing MDCK-Par1b cells. Shown is mean (green bar) and SEM (blue error bars). (B and C) Time-lapse analysis (stills from Movie S6) (B) and quantification (C) of control and MDCK-Par1b cells, indicating symmetric and asymmetric inheritance of apical plasma membrane domains in control and MDCK-Par1b cells, respectively. ***p<0.001. Scale bars: 5 µm. (TIF) [file pbio.1001739.s004.tif]

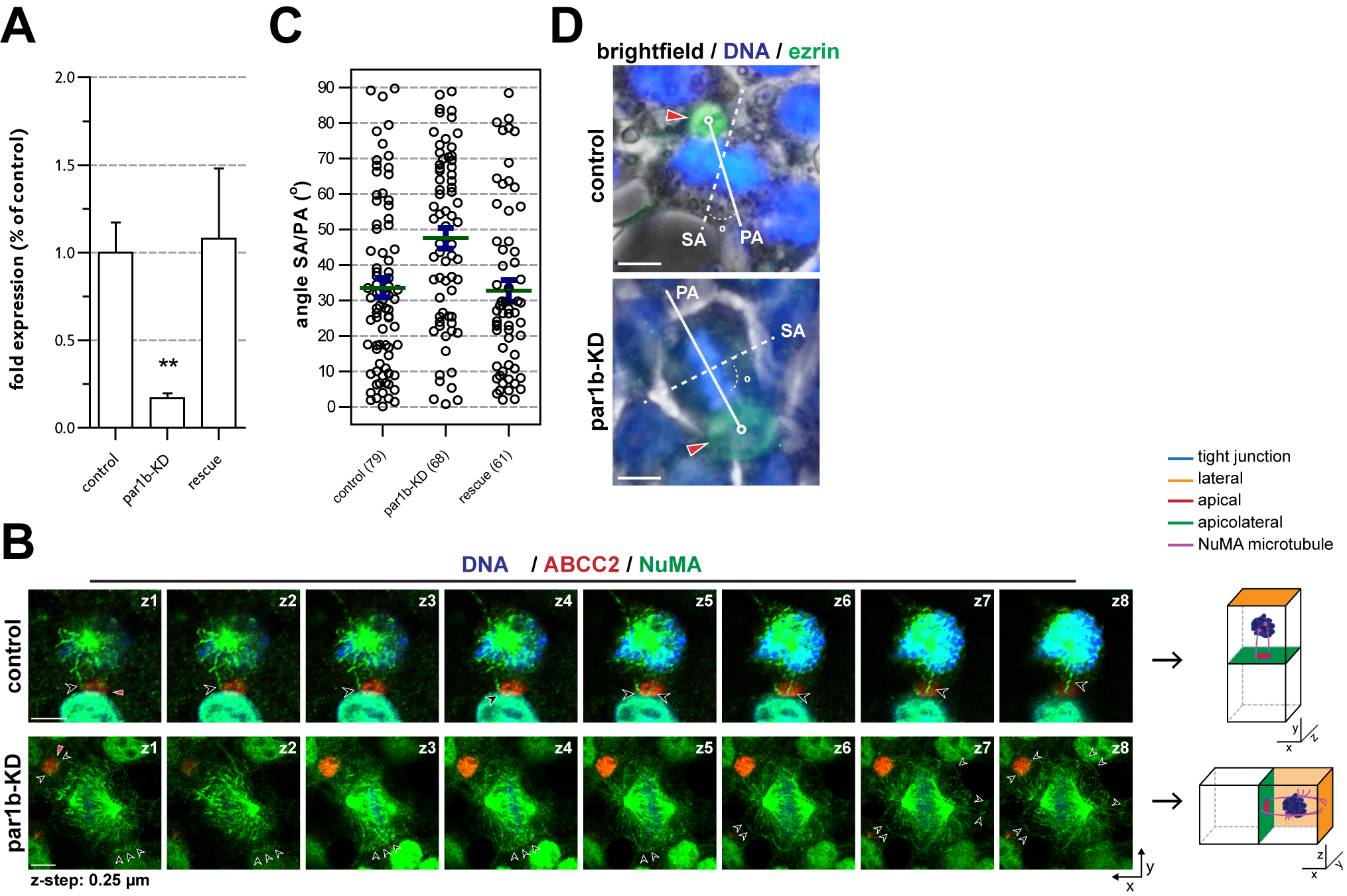

Supplement: Figure S5 — Par1b regulates apicolateral-directed spindle orientation in HepG2 cells. (A) Quantification of Par1b knockdown in HepG2 cells by quantitative PCR. (B) Stills from Movies S7 and S8, showing cortical NuMA (black arrowheads) at the apical domain and at both the apical and lateral membranes in control and Par1b knockdown HepG2 cells, respectively. (C) SA/PA angle was calculated for control (scrambled) shRNA and Par1b knockdown HepG2 cells (fixed) as indicated in (D) and plotted as depicted. All figures: red arrowheads mark the apical domain. The outlines show the identity of the cell membranes of the dividing cells (#) shown in (A). Red, orange, and green lines represent the apical, lateral, and apicolateral plasma membrane domains, respectively. **p<0.01. Scale bars: 5 µm. (TIF) [file pbio.1001739.s005.tif]

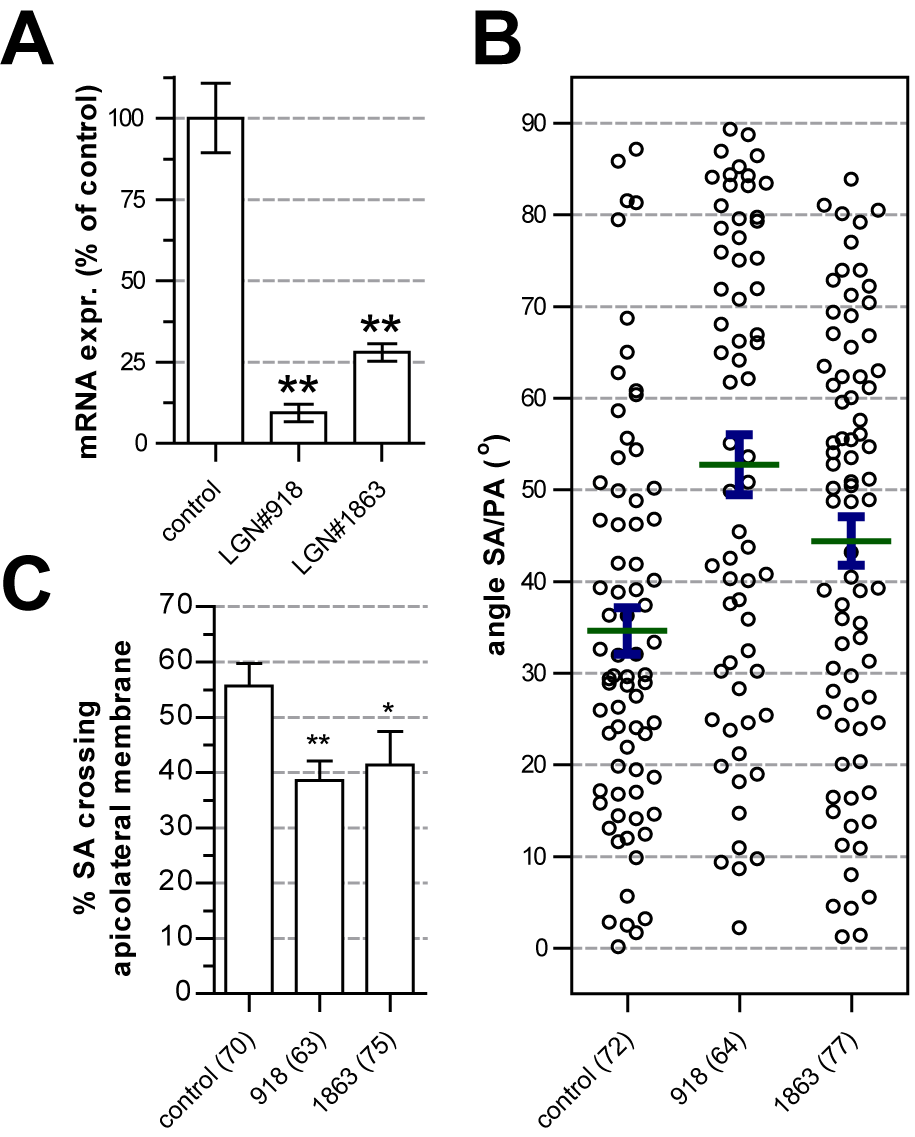

Supplement: Figure S6 — Short hairpin RNA targeted against LGN results in the depletion of the LGN protein in HepG2 cells, which, in turn, perturbs the apicolateral orientation of the mitotic spindle apparatus. (A) Real-time PCR analysis of the knockdown efficiency of the two LGN constructs used in this study. (B) Dot plot of SA/PA angles of HepG2 cells in metaphase under control and LGN knockdown conditions. Shown is mean with SEM. (C) Shown is the percentage of HepG2 cells with SA crossing the apicolateral membrane under control and LGN knockdown conditions, indicating reduced apicolateral spindle orientation under LGN knockdown conditions. *p<0.05. **p<0.01. (TIF) [file pbio.1001739.s006.tif]

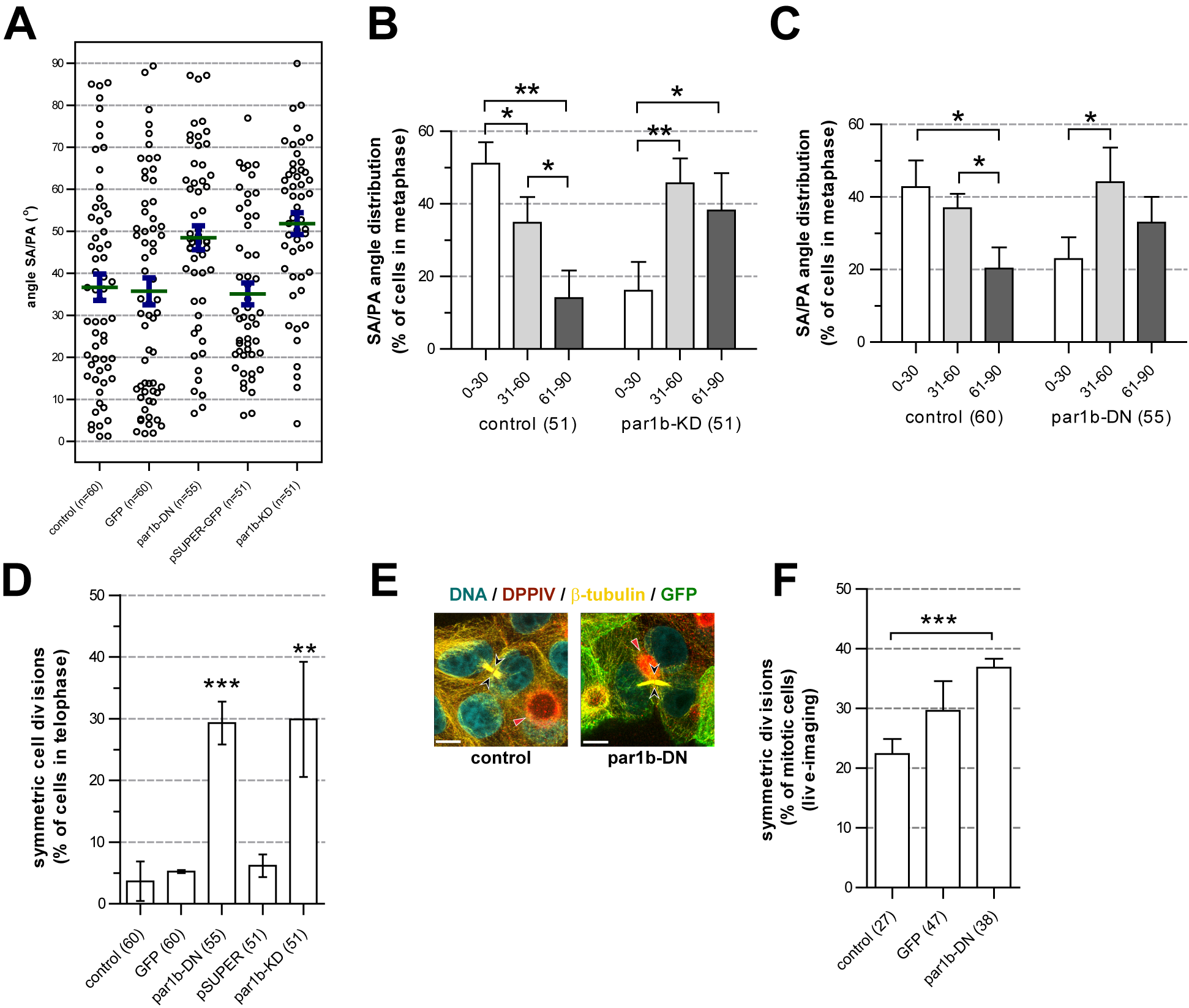

Supplement: Figure S7 — Knockdown of Par1b in WIF-B9 cells alters the orientation of the mitotic spindle apparatus relative to the apical polarity axis. (A) Dot blot showing individual SA/PA angles for dividing WIF-B9 cells under the depicted conditions. Shown is mean with SEM. (B) Histogram analysis of control (pSUPER) and Par1b-KD WIF-B9 cells indicating reduced apicolateral-subdomain-oriented spindle orientation (reduced bias towards lower angles [0–30°]) during Par1b depletion. (C) Histogram analysis of control (GFP) and Par1b-DN-GFP-expressing WIF-B9 cells indicating reduced apicolateral-subdomain-oriented spindle orientation (reduced bias towards lower angles [0–30°]) when Par1b function is perturbed. (D) WIF-B9 cells expressing Par1b-DN and Par1b knockdown cells in metaphase were scored for symmetric or asymmetric segregation of the apical plasma membrane. Reduced Par1b activity increased symmetric inheritance of the apical plasma membrane. (E) Illustrations of dividing control and Par1b-DN-expressing WIF-B9 cells. DPPIV marks the apical domain, β-tubulin marks the microtubules of the mitotic spindle. (F) Symmetry of cell division was also quantified during live imaging of WIF-B9 cells. Black arrowheads mark the ingressing cleavage furrow (midbody, site of cytokinesis). *p<0.05. **p<0.01. ***p<0.001. Scale bars: 5 µm. (TIF) [file pbio.1001739.s007.tif]
