# Supplementary material for: Par1b Induces Asymmetric Inheritance of Plasma Membrane Domains via LGN-Dependent Mitotic Spindle Orientation in Proliferating Hepatocytes
Source: PLoS Biol. 2013 Dec 17;11(12):e1001739. doi: 10.1371/journal.pbio.1001739 (PMC3866089; doi:10.1371/journal.pbio.1001739)
Supplement: Table S1 — A list of the commercial antibodies used in this study. (DOCX) [file pbio.1001739.s017.docx]

**Supplementary Table 1** | Commercial antibodies used in this study.

| **Target** | **Species** | **Company** | **Catalog#/Clone** |
| --- | --- | --- | --- |
| aPKC | rabbit | Santa Cruz Biotechnology | sc-216 |
| α-tubulin | rat | Abcam | Ab6160 |
| ezrin | rabbit | Santa Cruz Biotechnology | sc-20773 |
| abcc2 | mouse monoclonal | Enzo Life Sciences | ALX-801-016 |
| NuMA | rabbit | Abcam | ab36999 |
| ß-tubulin | mouse monoclonal | Millipore | MAB3408 |
| ZO-1 | mouse monoclonal | Life Technologies | clone 1A12 |
| DPPIV | mouse monoclonal | AbD Serotec | MCA924R |
| DPPIV | goat polyclonal | R&D Systems | AF954 |
