# Supplementary material for: Par1b Induces Asymmetric Inheritance of Plasma Membrane Domains via LGN-Dependent Mitotic Spindle Orientation in Proliferating Hepatocytes
Source: PLoS Biol. 2013 Dec 17;11(12):e1001739. doi: 10.1371/journal.pbio.1001739 (PMC3866089; doi:10.1371/journal.pbio.1001739)
Supplement: Table S2 — RNA interference target sequences for HepG2 cells. Listed are the sense sequences used to generate oligonucleotides according to the pLKO manual. (DOCX) [file pbio.1001739.s018.docx]

**Supplementary Table 2** | RNA interference target sequences for HepG2 cells. Listed are the sense sequences used to generate oligos according to the pLKO manual.

| **Target** | **Sequence** |
| --- | --- |
| Par1b | CAGCAAGAGAGGCACTTTAGA |
| LGN#918 | AAACGAATTCCTCCACTAAAG |
| LGN#1863 | AAACGAATTCCTCCACTAAAG |
