# Supplementary material for: Par1b Induces Asymmetric Inheritance of Plasma Membrane Domains via LGN-Dependent Mitotic Spindle Orientation in Proliferating Hepatocytes
Source: PLoS Biol. 2013 Dec 17;11(12):e1001739. doi: 10.1371/journal.pbio.1001739 (PMC3866089; doi:10.1371/journal.pbio.1001739)
Supplement: Table S3 — Real-time PCR primers used in this study. Listed are forward and reverse primer DNA sequences. (DOCX) [file pbio.1001739.s019.docx]

**Supplementary Table 3** | Real-time PCR primers used in this study. Listed are forward and reverse primer DNA sequences.

| **Target** | **Forward** | **Reverse** |
| --- | --- | --- |
| Par1b | GCAGCCCCACATTGGAAAC | CACAGCTACCTCTTTCCCAGTCA |
| HMBS | GGGAAACCTCAACACCCGGCT | ATCCTGGTTGTGCCAGCCCAT |
| GAPDH | CATTTCCTGGTATGACAACG | GTCCAGGGGTCTTACTCCTT |
| LGN | ATTAGTGACTGCTTTGGGTG | AGTGTCTTCTTGTAGTATTCCGAG |
